# Dietary calcium intake in relation to type-2 diabetes and hyperglycemia in adults: A systematic review and dose–response meta-analysis of epidemiologic studies

**DOI:** 10.1038/s41598-022-05144-8

**Published:** 2022-01-20

**Authors:** Zahra Hajhashemy, Parisa Rouhani, Parvane Saneei

**Affiliations:** 1 Students’ Research Committee, Isfahan University of Medical Sciences, Isfahan, Iran; 2 Department of Community Nutrition, School of Nutrition and Food Science, Isfahan University of Medical Sciences, PO Box 81745-151, Isfahan, Iran

**Keywords:** Nutrition, Diseases

## Abstract

Several epidemiological studies investigated the relation of Ca intake with type 2 diabetes mellitus (T2DM), but there were inconsistencies in their findings. So, we conducted a systematic review and dose–response meta-analysis to quantify the relation of dietary Ca intake with the risk of T2DM/hyperglycemia in adults. A systematic search was conducted up to May 2021, in MEDLINE (Pubmed), Web of Science (WOS), Scopus electronic databases and Google Scholar, for epidemiological studies that investigated the relation of dietary Ca intake (as the exposure) and T2DM/hyperglycemia (as the outcome) in adults, without restriction in publication date and language. Finally, 8 cohort and 9 cross-sectional studies were included in the analysis. The body of evidence was assessed by the GRADE approach. Combining effect sizes from prospective cohort studies included 255,744 general adult population illustrated that highest level of dietary Ca intake, compared to lowest category, was related to an 18% reduced risk of T2DM (RR: 0.82; 95% CI 0.74–0.92). Based on linear dose–response analysis (including 255,744 healthy individuals and 13,531 patients with T2DM), each 300, 600 and 1000 mg/day increment in dietary Ca intake was respectively associated to 7, 14 and 23% reduced risk of T2DM. There was a steeper reduction in risk of T2DM when dietary Ca intake increased from low levels to 750 mg/day. Nevertheless, meta-analysis of cross-sectional studies revealed an inverse significant association between dietary Ca intake and T2DM/hyperglycemia only in the female population (OR: 0.66; 95% CI 0.50–0.88). This meta-analysis illustrated an inverse association between dietary Ca intake and risk of T2DM in general adult populations in prospective cohort studies, in a dose–response manner. It seems that increasing dietary Ca intake from low levels to around 750 mg/day was inversely related to risk of T2DM. In cross-sectional studies, an inverse relation between dietary Ca intake and T2DM/hyperglycemia was found only in females.

## Introduction

Among the non-communicable diseases (NCDs), type 2 diabetes mellitus (T2DM) is the most prevalent with an increasing incidence at alarming rates worldwide^1^. T2DM could also drastically increase the risk of obesity, amputation, stroke, cardiovascular diseases (CVD), renal and liver diseases, and low quality of life^2–5^. Therefore, identification and management of T2DM modifiable risk factors are crucial steps in preventing and decreasing the incidence rate of this disease. Age, sex, genetic, race, lifestyle, physical activity, education and dietary intakes are important risk factors of T2DM^4–6^.

Several previous studies suggested that dietary nutrient intakes could be involved in blood glucose hemostasis. Calcium is a nutrient with a key role in the healthy development of bones and teeth; however, recent evidence documented non-skeletal functions for calcium. Intake of this nutrient could change the risk of stroke^7^, CVD^8^, colorectal and prostate cancer^9^, multiple sclerosis^10^, psoriasis, impaired glucose tolerance, insulin resistance, hyperglycemia and T2DM^11^. A recent systematic review has also suggested that Ca supplementation might have protective effects on low-density lipoprotein cholesterol (LDL-c) and high-density lipoprotein cholesterol (HDL-c) in overweight and obese subjects^12^.

Some previous studies documented that higher dietary Ca intake was significantly related to lower risk of T2DM in both men and women^13,14^. Nevertheless, some other investigations confirmed a significant relation only in men^15,16^ or could not find significant relation with T2DM^17–19^ or hyperglycemia^20–23^ in apparently healthy populations. Similarly, no significant relation was observed between dietary Ca intake and hyperglycemia or T2DM in patients with hypertension^24^ and renal transplant recipients^25^. Dong et al.^26^ have performed a systematic review and meta-analysis on 6 prospective studies published before 2012 and determined the relation between dietary Ca intake and T2DM in apparently healthy adults. They documented that Ca intake could be related to the risk of T2DM, but this relation was dependent on magnesium intake and further adjustment for magnesium intake made the association insignificant. They could not also perform subgroup analysis and meta-regression, due to low number of effect sizes (n = 7). After 2012, several other investigations on apparently healthy general adult populations have assessed this association. Most of these researches were prospective cohort studies^13,18,21,27–29^ and cross-sectional surveys or cross-sectional analysis of cohorts’ baselines with large populations^16,19,23,30,31^, but there were inconsistencies in their findings. So, we conducted a systematic review and meta-analysis to determine whether higher dietary calcium intake is associated with a lower risk of T2DM /hyperglycemia in adults. Dose–response analysis was also applied to examine the linear and non-linear relationship.

## Methods and materials

### Search strategy

All published articles were systematically searched in MEDLINE (Pubmed), Web of Science (WOS) and Scopus electronic databases as well as Google Scholar, up to May 2021. There was no restriction in the time of publication or language. Details of the applied MeSH and non-MeSH keywords in the systematic search are presented in Supplemental Table 1. Furthermore, we performed a manual search in bibliographies of the relevant investigations to identify additional studies. Grey literature including conference proceedings, unpublished articles and theses were not included in the present review. Two researchers (Z.H and P.R) independently performed all processes of the search strategy and the third investigator (P.S) supervised them.

The current analysis was performed according to the PRISMA checklist that is presented in Supplemental Table 2. We registered the study protocol at PROSPERO (CRD42021244394) (http://www.crd.york.ac.uk/Prospero).

### Inclusion criteria

All published papers were included in the current analysis if they: (1) had cohort, cross-sectional or case–control design; (2) investigated adult population (≥ 18 years), regardless of their health status; (3) considered dietary Ca intake as the exposure and reported the risk for abnormal glucose homeostasis including, T2DM, prediabetes or hyperglycemia as the outcomes of interest; (4) reported relative risks (RRs), hazard ratios (HR), or odds ratios (ORs), with 95% confidence intervals (CIs) for the association of dietary Ca intake and abnormal glucose homeostasis. The details of PICOS criteria (population, intervention/exposure, comparison/control, outcome, and study design) are presented in Table 1.

**Table 1:** PICOS criteria for inclusion of studies.

Parameter	Criteria
Participants	Adult population (≥ 18 years)
Intervention/Exposure	Different categories of dietary calcium intake
Control/Comparison	Individuals in the lowest category of dietary calcium intake
Outcome	Abnormal glucose homeostasis including, type-2 diabetes, prediabetes and hyperglycemia
Study design	Observational studies including prospective cohort, cross-sectional and case–control studies

In this systematic review and meta-analysis, we considered abnormal blood glucose homeostasis as the primary outcome. Included studies have used various validated methods and cut-off-points for their populations to define abnormal blood glucose homeostasis. They categorized abnormal blood glucose homeostasis as T2DM, prediabetes or hyperglycemia. Therefore, we considered T2DM, prediabetes or hyperglycemia as the main outcomes of interest.

### Exclusion criteria

Supplemental Table 3 contains details of more relevant studies that were not included in the current study. Investigations were excluded if they: (1) standard or un-standard regression coefficient (β or B), reported correlation coefficient, mean ± SE or mean ± SD or median (Inter Quartile Range) for blood glucose across categories of dietary Ca intake; (2) considered change in blood glucose concentrations as the outcome of interest; (3) considered type 1 diabetes (T1D) or gestational diabetes mellitus (GDM) as the outcome; (4) considered hyperglycemia or diabetes as the exposure and dietary Ca intake as the outcome. In addition, studies with overlapping study population were excluded. We found 2 reports from the following investigations: National health and nutrition examination surveys (NHANES 2001–2014)^19,31^ and Multi-Rural Communities Cohort (MRCohort 2005–2011)^18,28^. Therefore, only the publication with higher sample size from each pair of these investigations (NHANES 2007–2014^19^, MRCohort 2005–2011^18^) was included in the analysis.

### Data extraction

The essential information independently extracted by two investigators (Z.H, P.R) was the first authors’ last name, study location, study design, year of publication, longitude, latitude, sample size, recruitment source of population, health status of participants, development status of the countries, mean age, sex, representativeness of the study population, method of dietary Ca intake assessment, Ca intake levels, prevalence of hyperglycemia in dietary Ca intake levels, RRs, HRs or ORs with 95% CIs for T2DM or hyperglycemia, definition of T2DM or hyperglycemia, method of serum blood glucose measurement and adjustments for confounders. Notably, the most fully adjusted risk estimates were extracted. In addition, for dose–response analysis, we extracted the number of cases with T2DM or hyperglycemia, total number of participants and the mean values of Ca intake in each category of dietary Ca intake.

### Quality assessment of studies

We used the Newcastle–Ottawa Scale (NOS)^32^ to determine the quality score of studies. Based on NOS a maximum 9 score was given to each cohort investigation, including 5 for individual selection (demonstration that T2DM or hyperglycemia was not present at the start of the study, selection of the non-exposed cohort, ascertainment of dietary Ca intake as the exposure, representativeness of the exposed cohort), 2 scores for comparability (adjustment for confounders including, age and sex), and 3 scores for assessment of the outcome (validated assessment of T2DM or hyperglycemia, adequacy of follow-up of cohorts and enough duration of follow-up for incidence of T2DM or hyperglycemia). Moreover, a maximum of 10 score was assigned for each cross-sectional study. Five scores for participant selection (representativeness of study population, satisfaction of sample size and ascertainment of dietary Ca intake as the exposure, description of non-respondents), 2 scores for comparability (controlling for confounders including, age and sex), and 3 scores for the outcome (validated assessment of T2DM or hyperglycemia and using an appropriate statistical test for the analysis). Details of quality assessment of eligible studies are presented in Supplemental Table 4. In the current meta-analysis, studies with a quality score of 8 or more were determined as "high quality" and other studies were deemed to be "low quality". Moreover, Grading of Recommendations, Assessment, Development and Evaluations (GRADE) was used to determine the quality of evidence^33^ through GRADEpro (GRADEproGDT, www.gradepro.org^34^. According to this approach, we evaluated the main factors that could downgrade the study quality including indirectness of evidence, risk of bias, inconsistency of findings, imprecision of findings, and publication bias. The factors upgrading quality were also considered through the evaluation of dose–response analysis, large effect and plausible confounding. Based on GRADE approach, the certainty of the body of evidence could be rated in one of four categories: high, moderate, low and very low. Results of GRADE assessment of this meta-analysis are presented in Supplemental Table 5.

### Statistical analysis

We used the reported RR/HR/OR and 95% CI for dietary Ca intake in relation to T2DM or hyperglycemia to compute the log RR/HR/OR and its standard error. In order to calculate the overall effect size, the random-effects model that takes between-study variation into account was applied. In order to indicate heterogeneity, we calculated Cochran's Q test as well as I^2^. When heterogeneity was significant, in order to examine its source, we performed subgroup analyses based on several confounders including sex, age (< 50 vs. ≥ 50 years), Ca intake assessment tools, quality of studies, study location (Asian vs. Non-Asian countries), developmental status of the countries, methods of blood glucose measurement, representativeness of study population, and health status of participants. We performed a fixed-effect model to determine between subgroups heterogeneity. Utilizing meta-regression, we also tested the influence of continuous variables (including latitude, quality of studies, longitude and age) on the overall estimate. To assess whether each study could influence the results, sensitivity analysis was applied. Publication bias was examined visually by inspecting funnel plot asymmetry, as well as using statistical tests of Egger's and Begg's. According to the methods previously described by Longnecker^35^ and Orsini et al.^36^, dose–response analysis was applied by using RRs/HRs/ORs and their 95% CIs, number of cases with T2DM or hyperglycemia, total number of individuals and median or mean level of dietary Ca intake in each category. When an investigation reported the range of Ca intake, we computed the midpoint by averaging the lower and the upper bounds. In studies that reported an open-ended highest category, we considered the length of interval as the same as the adjacent category. In case of the open-ended lowest category, the lower boundary was considered zero and the mean level of dietary Ca intake was calculated. The specific slopes (linear trends) and 95% CIs for 300, 600 and 1000 mg/day dietary Ca intake were computed through the use of natural logs of the RRs/HRs/ORs and their 95% CIs across dietary Ca levels. Non-linear dose–response analysis required studies with at least 3 different categories of dietary Ca intake. Non-linear relation between dietary Ca intake and T2DM or hyperglycemia was assessed using the restricted cubic splines (considering three knots at fixed percentiles of 10%, 50% and 90% of the distribution). We applied statistical analyses through the use of statistical software package Stata version14.0. P values less than 0.05 were considered statistically significant.

## Results

**Figure 1:**
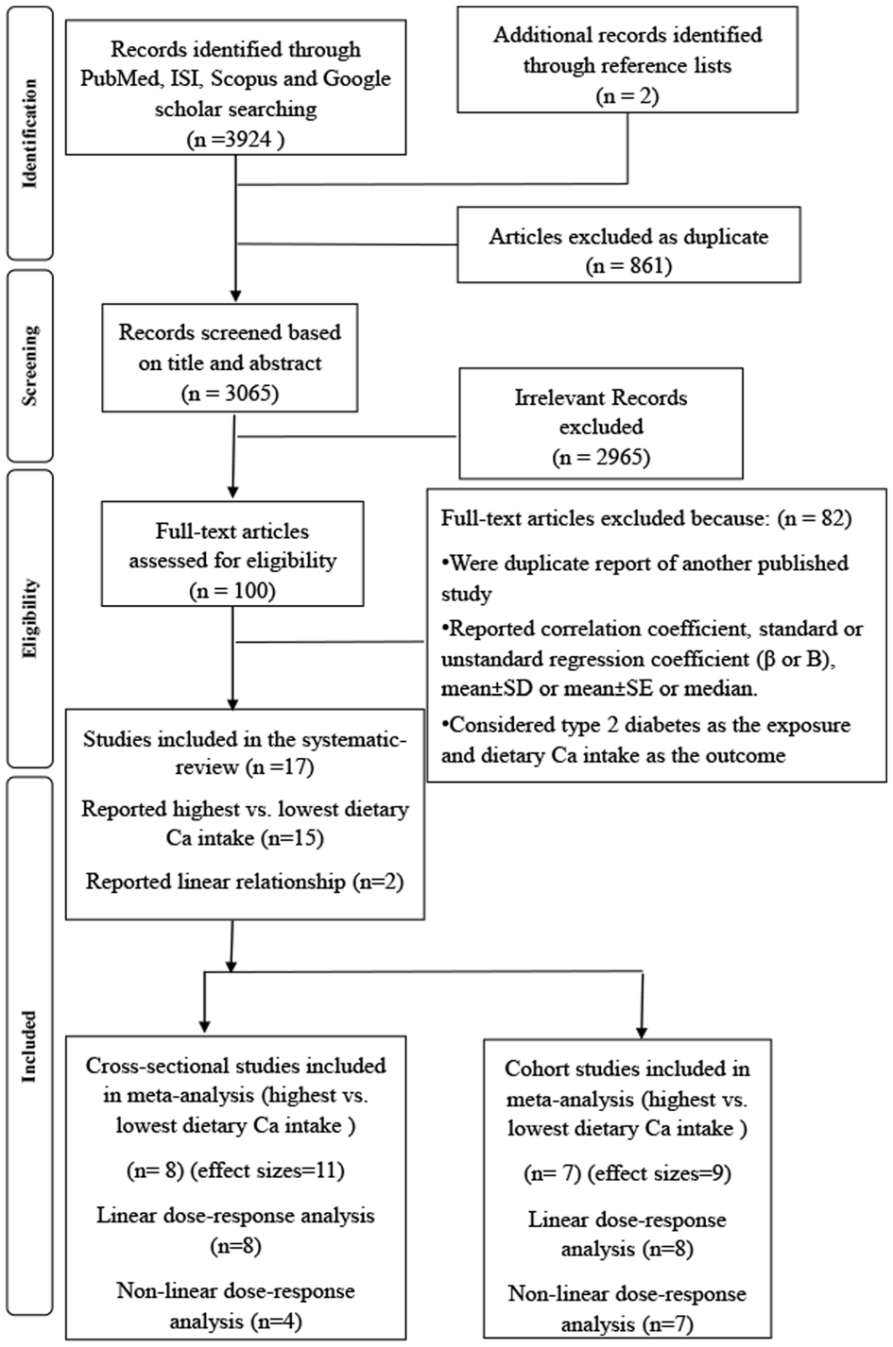
Flow diagram of search strategy and study selection.

Overall, 3924 publications were obtained in our comprehensive systematic search. Additionally, 2 papers were found through the manual search of reference lists. Then, 861 duplicate studies were excluded. In the primary round, the title and abstract of 3065 reports were screened and 2965 investigations were excluded. In the secondary round, the full-text of 100 reports was carefully investigated and after excluding 82 irrelevant investigations, 17 eligible studies were included in the systematic review and meta-analysis, as shown in Fig. 1.

### Study characteristics

Overall, 17 epidemiologic investigations published between 2006 and 2021 were included in this systematic review. Details of these eligible investigations are presented in Table 2. Eight prospective cohorts^13,14,17,18,21,37–39^ and 9 cross-sectional studies^15,16,19,20,22–25,30^ had investigated the relationship between dietary Ca intake and abnormal glucose homeostasis among 301,116 individuals. Four eligible studies were conducted in USA^19,21,37,38^ and 4 others in Korea ^15,16,18,30^, 3 in Brazil^22,24,25^, 2 in China^13,14^ and 2 in Australia^17,23^ and the remaining in Turkey^20^ and Japan^39^ (eight studies in Asian countries and 9 others in non-Asian regions). The assessment of dietary Ca intake was performed through the use of food frequency questionnaires (FFQs) in 12 reports, food recall in 4 investigations and food record in the last study. Nine of included studies considered T2DM as the outcome and the others reported high blood glucose (HBG) or hyperglycemia (fasting blood glucose (FBG) ≥ 100 or 110 mg/dL) as the outcome of interest. Fifteen studies investigated generally healthy populations and just 2 cross-sectional investigations were conducted on patients with hypertension^24^ and renal transplant recipients^25^. Thirteen investigations have used random sampling method to select their participants, so their subjects were representative of general adult populations; while 4 others did not randomly select their individuals. Fifteen of 17 studies were included in the meta-analysis of highest vs. lowest dietary Ca intake in relation to T2DM/hyperglycemia, while the two remaining investigations^21,23^ (one cohort and one cross-sectional study) that reported linear relationships, were only included in the linear dose–response analysis. Among the included studies, 12 studies received a score of 8 or more; therefore, they were classified as high-quality studies, while the remaining studies were classified as low-quality.

**Table 2:** Main characteristics of included studies examined the association between dietary Ca intake and T2DM/hyperglycemia.

First Author & (Year)/Ref	Study design/ Cohort name	Country	Age Range/ mean ± SD	Gender/female%	No. Participants/ No. Case	Exposure assessment	Calcium intake levels, mg/day	OR, HR or RR (95%CI)	Outcome definition	Outcome assessment	Health status of participants	Adjustments	score
Shah (2021)/^19^	Cross-sectional (NHANES 2007–2014)	USA	47.9 ± 5.1	M	9977/1775	24-h dietary recall	Mean Q5: (1538) vs. Q1: (397)	OR (95%CI) 1.2 (0.80, 1.80)	T2D (ADA 2014: A1C ≥ 6.5% and/or fasting glucose ≥ 7 mmol/L and/or diagnosed T2DM)	NR	Adults	1–11	8
				F 51.3%	10,503/1657		Mean Q5: (1457) vs. Q1: (369)	OR (95%CI) 1.0 (0.70, 1.50)					
Talaei (2018)/^13^	Cohort Singapore Chinese Health Study (1999–2010)	China	45-74y /55.2	M/F 57.3%	45,411/5207	Validated FFQ	Median Q4: (597) vs. Q1: (258)	HR 0.75 (0.66, 0.85)	T2D (Self-reported history of physician-diagnosed T2D)	NR	Adult	1, 2, 4–8, 10–14, 16–18, 22–24, 32, 34, 43	9
Aritici (2018)/^20^	Cross-sectional	Turkey	19–52 /34.59	F	146/14	3-day self-reported nutrient intake	Highest vs. Lowest	OR (95%CI) 0.43 (0.94–2.19)	HBG (plasma glucose ≥ 110 mg/dL or the use of antidiabetic medicine/insulin)	Autoanalyzer	Premenopausal women	1, 2, 6–8	7
Beydoun (2018) /^21^	Cohort HANDLS (2004–2009 and 2009–2013)	USA	48.6 ± 0.4	M	557/NR	24-h dietary recall	Per 821.9 mg increment in Ca intake	HR 0.79 (0.37, 1.67)	HBG (FBG ≥ 110 mg/dl)	Immuno-Analyzer	Urban adults	1–3, 7–10, 22,24, 26–31, 33–35, 39, 43, 58–62	7
			48.2 ± 0.3	F 59.4%	814/NR		Per 659.7 mg increment in Ca intake	1.03 (0.87, 1.22)					
Oh, (2017)/^18^	Cohort MRCohort 2005–2011	Korea, Yangpyeong	≥ 40	M	3011/128	Validated FFQ & 12-days 24-h dietary record	Median T3: (462) vs. T1 (197)	IRR 0.73 (0.45,1.17)	T2D (ADA: FBG ≥ 126 mg/dL or treatment with oral hypoglycemic medication or insulin.)	Automatic Analyzer	Adults	1, 5, 6, 10,11,17, 45	8
				F 62.6%	5048/187		Median T3: (458) vs. T1: (180)	0.61 (0.43,0.87)					
Pannu (2017)/^23^	Cross-sectional (VHM)	Australia	18-75y	M/F 53.4%	3387/1811	Five-pass 24-h diet recall	Per 500 mg increment in Ca intake	OR (95%CI) 0.99 (0.77, 1.26)	HBG (FBG ≥ 100 mg/dl)	Hexokinase method	Adults	1, 2, 4–10, 17, 19, 21, 36, 46–48, 50	9
Shin (2016)/^30^	Cross-sectional (KNHANES 2010–2012)	Korea	44.4 ± 0.2	M	5946/1789	24-h recall and 63-item FFQ	Range Q4: (716.7–5049.1) vs. Q1: (30.9–333.8)	OR (95%CI) 1.10 (0.75, 1.61)	HBG (FBG ≥ 100 mg/dL or use of medication to treat diabetes mellitus)	Autoanalyzer	Obese males	1, 4–10, 20, 46, 49, 50	10
Shin(2015)/^16^	Cross-sectional (KoGES) Part of (MRCohort) 2005–2010	Korea Yangpyeong, Namwon and Goryeong	61.5 ± 9.8	M	2331/917	Validated FFQ	Median Q4: (525.4) vs. Q1: (188.4)	OR (95%CI) 1.02 (0.72, 1.39)	HBG (FBG ≥ 100 mg/dL or use of medication to treat diabetes mellitus)	Automatic Analyzer	Adults (calcium and multi-nutrient non-users)	1, 5, 36, 39, 41, 45	9
			59.7 ± 10	F 59.8%	3473/930		Q4 (506.9) vs. Q1 (161)	0.66 (0.48, 0.90)				1, 5, 6, 10, 36, 39, 41, 45, 55, 56	
Ferreira (2013)/^22^	Cross-sectional	Brazil	18-50y 31.3 ± 1.3	F	76/4	Validated FFQ	Highest (≥ 600)vs. Lowest(< 600)	OR (95%CI) 0.23 (0.01, 3.85)	HBG (FBS ≥ 1000 mg/l)	RIA	Healthy pre-menopausal women	1, 8, 10, 37, 38, 41	9
Gagnon (2011)/^17^	Cohort (AusDiab 1999–2005) (5y)	Australia	50.7 ± 12.5	M/F 54.7%	5200/199	Validated FFQ	Q4: (1060–2317) vs. Q1: (171–740)	OR (95%CI) 0.94 (0.61, 1.46)	T2D (treatment with insulin or oral hypoglycemic agents, FPG ≥ 7 mmol/L or 2-h plasma glucose (PG) post-OGTT ≥ 11.1 mmol/L	Glucose oxidase method	Adults	1, 3, 7, 10, 14, 15, 17, 19, 44, 47, 51, 53, 64	7
Torres (2011)/^25^	Cross-sectional	Brazil	> 18/47.3	M/F 46%	74/8	24-h recalls	Highest (≥ 600)vs. Lowest(< 600)	OR (95%CI) 0.12 (0.004–3.16)	T2D (FBS ≥ 126 mg/dL or using insulin or an oral antidiabetic for a minimum of 8 weeks)	NR	Renal transplant recipients	1, 2, 6, 10, 62, 63	7
Torres (2011)/^24^	Cross-sectional	Brazil	25-70y /56.9 ± 1.3	M/F 80.7%	57/18	Validated FFQ	Highest (≥ 800)vs. Lowest(< 800)	OR (95%CI) 1.07 (0.96, 1.19)	HBG (FBG ≥ 100 mg/dl)	Glucose oxidase method	Hypertensive patients	1, 2, 4, 6, 10, 51, 52	7
Kim (2012)/^15^	Cross-sectional ((KARE) part of the KoGES)	Korea	51.8 ± 0.2	M	3846/690	Validated FFQ	Mean T3 (567.2) vs. T1 (282.9 mg)	OR (95%CI) 1.06 (0.81, 1.37)	HBG (FBS ≥ 100 mg/dL or treatment of T2D)	NR	Adults	1, 5, 6, 7(M), 10, 36, 37, 39, 40, 45	10
				F 52.1%	4185/456		Mean T3: (628.7) vs. T1: (287.6)	0.68 (0.50, 0.92)					
Kirii (2009)/^39^	Cohort (JPHC) (5y)	Japan	40-69y 56.9 ± 0.8	M	25,877/634	Validated FFQ	Median Q4: (629) vs. Q1: (254)	OR (95%CI) 0.93 (0.71, 1.22)	Self-reported T2D that validated by (FBG ≥ 7.8 mmol/l; and casual plasma glucose ≥ 11 mmol/l)	NR	Middle-aged and older population	1, 4, 6–8, 10, 14, 15, 17, 32, 46	8
				F 56.7%	33,919/480		Q4: (810) vs. Q1: (356)	0.76 (0.56, 1.03)					
Villegas (2009)/^14^	Cohort (SWHS) (6.9)	China	40-70y 50.4 ± 8.6	F	64,190/2270	Validated FFQ	Q5 (649.6) vs. Q1 (227.5)	RR (95%CI) 0.74 (0.65, 0.85)	T2D (FBG ≥ 7 mmol/L on 2 ≥ separate occasions or an OGTT ≥ 11.1 mmol/L and/or use of hypoglycemic medication)	NR	Women	1, 4–10, 14, 54	9
Van Dam(2006)/^37^	Cohort Black Women’s Health Study 1995–2003 (8y)	USA	21-69y 38.7 ± 0.2	F	41,186/1964	Validated FFQ	Q5: (661) vs. Q1: (219)	HR 1.04 (0.88, 1.24)	T2D (Self-reported, validated by physicians’ diagnosis)	-	Black women	1, 4–8, 10, 15, 17, 24, 25, 32, 35	9
Pittas(2006)/^38^	Cohort NHS 1980–2000 (20y)	USA	45.9 ± 0.3	F	31,901/2465	Validated FFQ	Q4: (> 1000) vs. Q1: (≤ 500)	RR (95%CI) 0.92 (0.76, 1.12)	T2D ((FBG ≥ 7.8 mmol/l or randomly measured plasma glucose ≥ 11.1 mmol/l)	NR	Women	1, 4, 6–8, 14, 15, 20, 33, 46	8

1-Age, 2-gender, 3-race, 4-BMI, 5-education level, 6-exercise, 7-smoking status, 8- alcohol use, 9-economic status, 10- energy intake, 11-vitamin D intake, 12- dialect, 13-year of interview, 14-hypertension, 15-diabetes, 16-potasium intake, 17-mangasium, 18-phosphorius, 19-calcium, 20-multivitamins, 21-zinc, 22-vegetable intake, 23-fruit, 24-meat, 25-processed meat, 26-poultry, 27-fish, 28-nut, 29-seed, 30-grains, 31-legums, 32-coffee, 33-caffeine, 34-soda, 35-sugar-sweetened drink, 36-fiber, 37-protein, 38-carbohydrate, 39-fat, 40-cholestrol, 41-sodium, 42-polutary, 43-soy, 44-FBG, 45-glycemic load, 46-residential area, 47-season, 48-MetS components, 49-eGFR, 50–25(OH)D levels, 51-WC, 52-anti-hypertensive agents, 53-latitude, 54-waist-hip ratio, 55-marital status, 56-farmer, 57-supplement use, 58-drug, 59-egg consumption, 60-oils, 61-health status, 62-time from transplantation, 63-dose of prednisone, 64-TG levels.

Ref, reference; SD, Standard Deviation; NO, number; OR, odds ratio; HR, hazard ratio; RR, relative risk, IRR, incidence rate ratio; Y, year; M, male; F, female; g, gram; d, day; T, tertiles; Q, quartiles; BMI, body mass index; SBP, systolic blood pressure; DBP, diastolic blood pressure; YAQ, Youth Adolescent Food Frequency Questionnaire; HS-FFQ, Harvard Service Food Frequency Questionnaire; WC, waist circumference; FBG, fasting blood glucose; JPHC, Japan Public Health Center; HBG, high blood glucose; MRCohort, Multi-Rural Communities Cohort; Mg, magnesium; Ca, calcium; T2B, type 2 diabetes; HBG, high blood glucose; IFG, Impaired fasting glucose; KoGES, Korean Genomic Epidemiology Study; VHM, Victorian Health Monitor; SQFFQ, semi quantitative FFQ; NHNES, National Health and Nutrition Examination Survey; KARE, Korea. Association Resource; J-MICC, Japan Multi-Institutional Collaborative Cohort; KoGES, Korean Genome and Epidemiology Study; VHMS, Victorian Health Monitor survey, ADA, American Diabetes Association; HANDLS, Healthy Aging in Neighborhoods of Diversity Across the Life Span; OGTT, oral-glucose-tolerance test; NHS, Nurses’ Health Study; FPG, fasting plasma glucose; TG, Triglyceride; M, male.

### Findings from meta-analysis of highest vs. lowest dietary Ca intake and T2DM in prospective cohort studies

**Figure 2:**
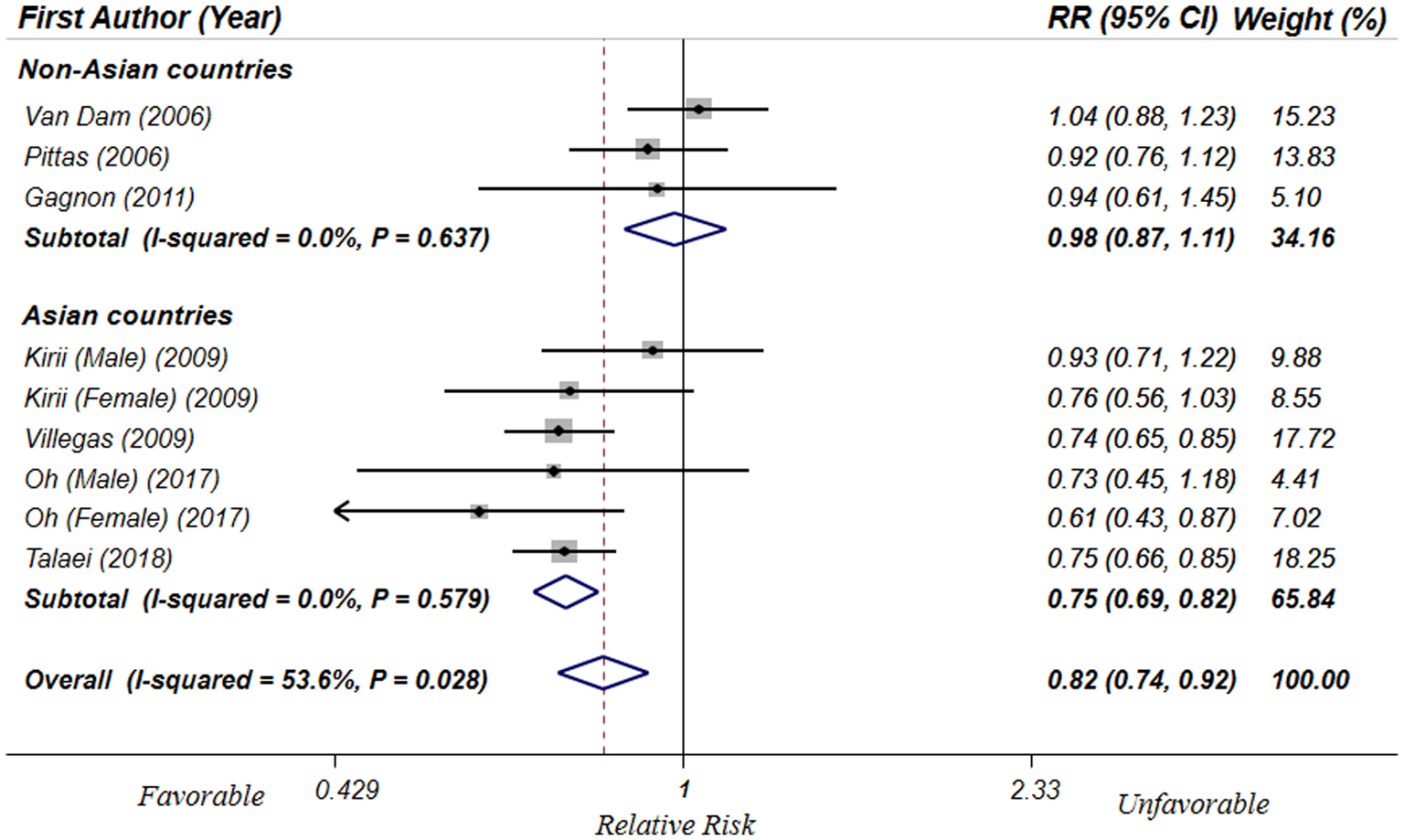
Forest plots of the association of highest vs. lowest level of dietary Ca intake and T2DM in prospective cohort studies with representative adult populations, stratified by study location (Asia vs. Non-Asia).

In total, 7 cohort studies^13,14,17,18,37–39^ with representative populations (including 255,744 individuals) were included in this analysis. Results revealed that individuals in the highest category of Ca intake, in comparison with the lowest one, had 18% lower risk of T2DM (RR: 0.82; 95% CI: 0.74, 0.92) (Fig. 2). Nevertheless, the heterogeneity between studies was moderate (I^2^ = 53.6, P_Q-test_ = 0.02). Therefore, subgroup analysis was conducted based on study location (Asian vs. non-Asian countries); between-study heterogeneity was removed in both Asian (I^2^ = 0.0, P_Q-test_ = 0.57) and non-Asian (I^2^ = 0.0, P_Q-test_ = 0.63) subgroups. However, the Ca intake-T2DM relation was significant only in Asian countries (RR: 0.75; 95% CI: 0.69, 0.82) and there was no significant relation in non-Asian regions (RR: 0.98; 95% CI: 0.87, 1.11) (Fig. 2). Further subgroup analyses were also conducted based on other confounders, and the results are shown in Table 3. Higher dietary Ca intake was protectively associated with lower risk of T2DM in subgroups of females, high-quality studies, investigations with adjustment for Mg intake, among participants with mean age ≥ 50 and in developing countries. However, heterogeneity was still significant in some of these subgroups. Additionally, meta-regression was applies and showed that mean age of participants (β = − 0.014, P = 0.02, I^2^_residual_ = 0.0%) and the latitude of study location (β = 0.024, P = 0.03, I^2^_residual_ = 17.92%) would contribute to the overall estimate; nevertheless, longitude (β = −0.002, P = 0.40, I^2^_residual_ = 51.51%) and quality of included studies (β =  − 0.019, P = 0.85, I^2^_residual_ = 58.01%) did not have significant effect on the pooled RR. Furthermore, sensitivity analysis showed that none of the eligible studies influenced the results. Based on funnel plot symmetry and results of Begg’s test (P = 0.67) and Egger’s test (P = 0.83), there was no evidence for publication bias.

**Table 3:** Results of subgroup-analyses of dietary Ca intake in relation to T2DM in prospective cohort studies.

	No. of effect sizes	RR (95% CI)	P within^1^	I^2^ (%)	P between^2^
Overall	9	**0.82 (0.74, 0.92)**	0.02	53.6	
Sex					0.41
Male	2	0.88 (0.69, 1.11)	0.38	0.0	
Female	5	**0.82 (0.69, 0.98)**	0.00	71.0	
Both	2	**0.76 (0.68, 0.86)**	0.33	0.0	
Adjustment for Mg intake				0.51	
Yes	7	**0.83 (0.71, 0.96)**	0.03	56.0	
No	2	0.82 (0.66, 1.01)	0.07	69.5	
Mean age					0.00
< 50	2	0.99 (0.87, 1.12)	0.35	0.0	
≥ 50	7	**0.76 (0.70, 0.82)**	0.57	0.0	
Development status of study location			0.00		
Developed	7	0.87 (0.77, 1.00)	0.14	36.7	
Developing	2	**0.75 (0.68, 0.82)**	0.88	0.0	
Quality score^3^					0.53
Low quality (Scores ≤ 7)	1	0.94 (0.61, 1.45)	-	-	
High quality (Scores > 7)	8	**0.82 (0.73, 0.92)**	0.01	58.4	
Reported Estimates					0.40
HR	2	0.88 (0.64, 1.21)	0.00	88.9	
IRR and RR	4	**0.77 (0.65, 0.90)**	0.15	43.3	
OR	3	0.87 (0.72, 1.04)	0.57	0.0	

^1^P for heterogeneity, within subgroup.

^2^P for heterogeneity, between subgroups.

^3^Quality Scores were according to Newcastle–Ottawa Scale.

### Findings from dose–response meta-analysis of dietary Ca intake and T2DM/hyperglycemia in prospective studies

**Figure 3:**
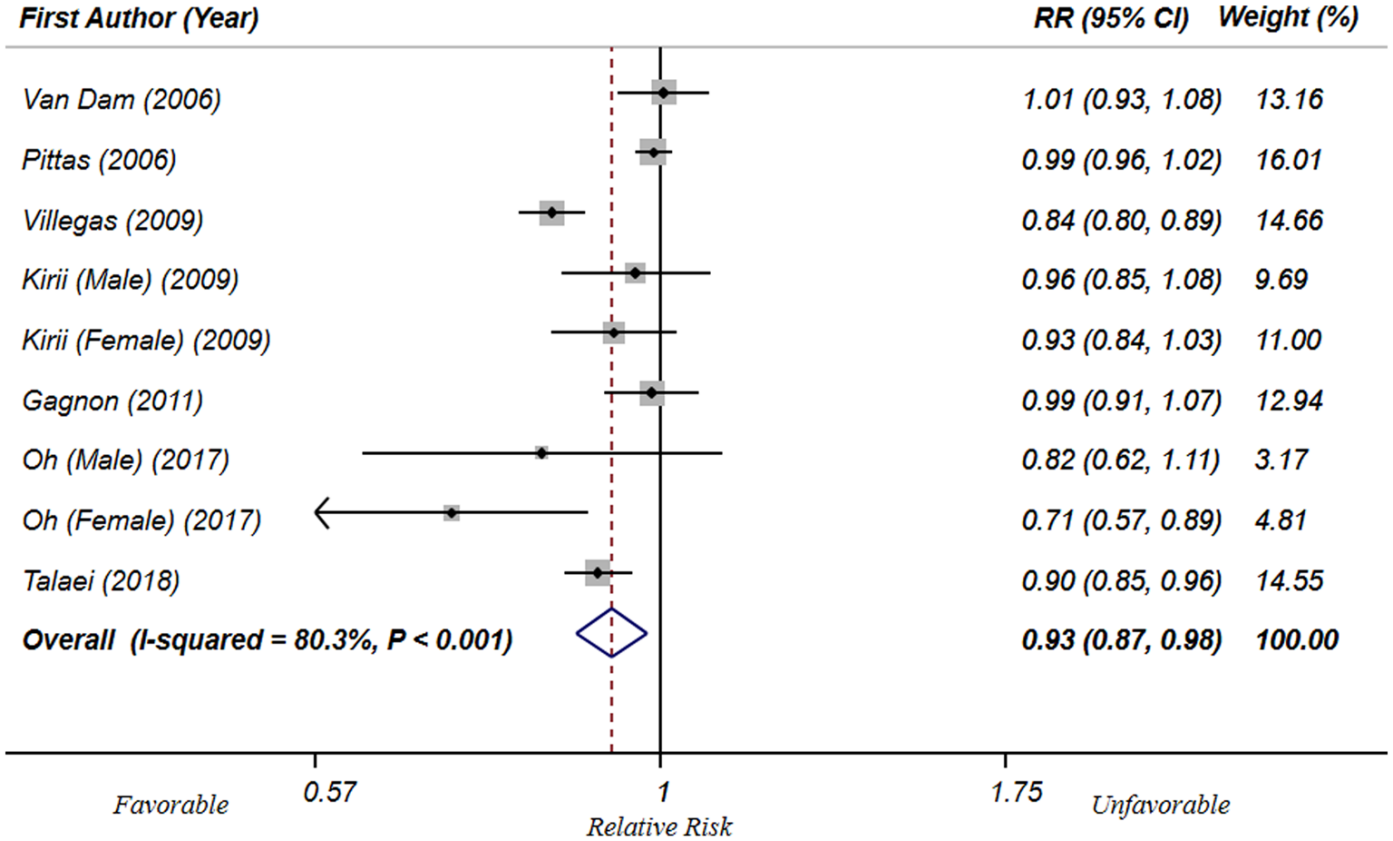
Forest plots of linear dose–response meta-analysis of the association between each 300 mg/day increment in dietary Ca intake levels and T2DM in prospective cohort studies with representative adult populations.

**Figure 4:**
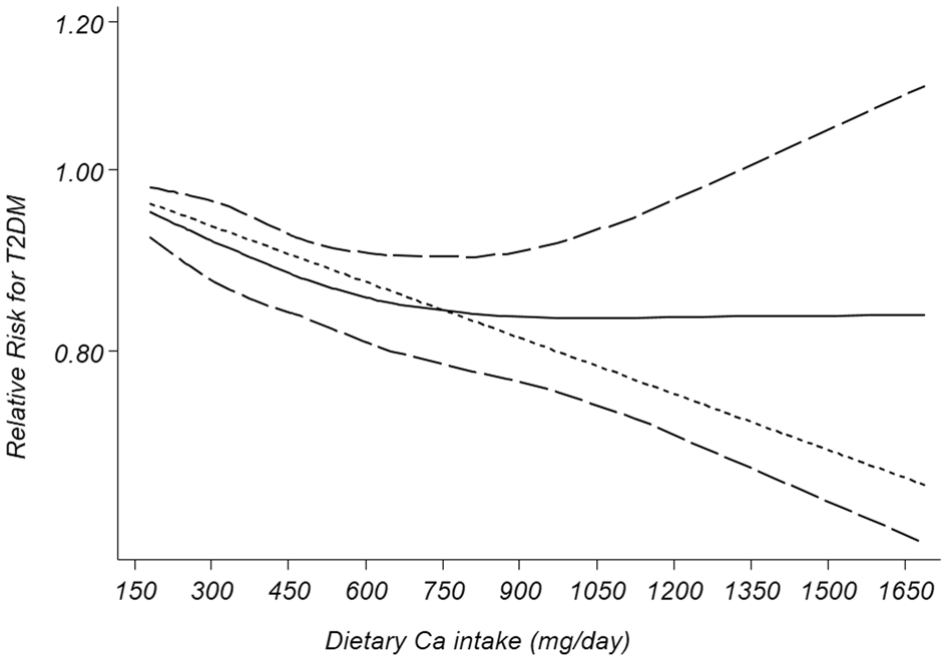
Non-linear dose–response association between dietary Ca intake levels and T2DM in prospective cohort studies with representative adult populations.—-—, Linear model; ____, spline model.

Eight cohorts with representative populations were eligible for the linear dose–response analysis^13,14,17,18,21,37–39^. Findings showed that each 300, 600 and 1000 mg/d increase in dietary Ca intake was significantly related to a 7%, 13% and 20% decreased risk of T2DM/hyperglycemia (RR: 0.93; 95% CI 0.89, 0.98), (RR: 0.87; 95% CI 0.79, 0.97) and (RR: 0.80; 95% CI 0.67, 0.95) , respectively. Then, we excluded the study of Beydoun et al.^21^ that considered hyperglycemia as the outcome; the analysis of 7 cohort studies^15,16, 28–32^ (including 255,744 participants with 13,531 cases of T2DM) revealed that each 300 mg/day increment in dietary Ca intake were significantly related to 7% decreased risk of T2DM (RR: 0.93; 95% CI 0.87, 0.98) (Fig. 3). In addition, each 600 and 1000 mg/d increase in dietary Ca intake were significantly related to 14% and 23% decreased risk of T2DM [(RR: 0.86; 95% CI 0.76, 0.96) and (RR: 0.77; 95% CI 0.64, 0.94), respectively. (Supplemental Figs. 1 and 2). Furthermore, non-linear dose–response analysis on 7 prospective cohort sudies^13,14,17,18,37–39^ (including 255,744 participants with 13,531 cases of T2DM) showed no significant association between dietary Ca intake and T2DM (P _non-linearity_ = 0.21) (Fig. 4). The threshold effect of dietary Ca intake was about 750 mg/day; such that, there was a steeper reduction in risk of T2DM when dietary Ca intake increased from low levels to 750 mg/day. Nevertheless, at Ca intakes above 750 mg/day, the risk was not reduced anymore.

### Findings from meta-analysis of highest vs. lowest dietary Ca intake and T2DM/hyperglycemia in cross-sectional studies

**Figure 5:**
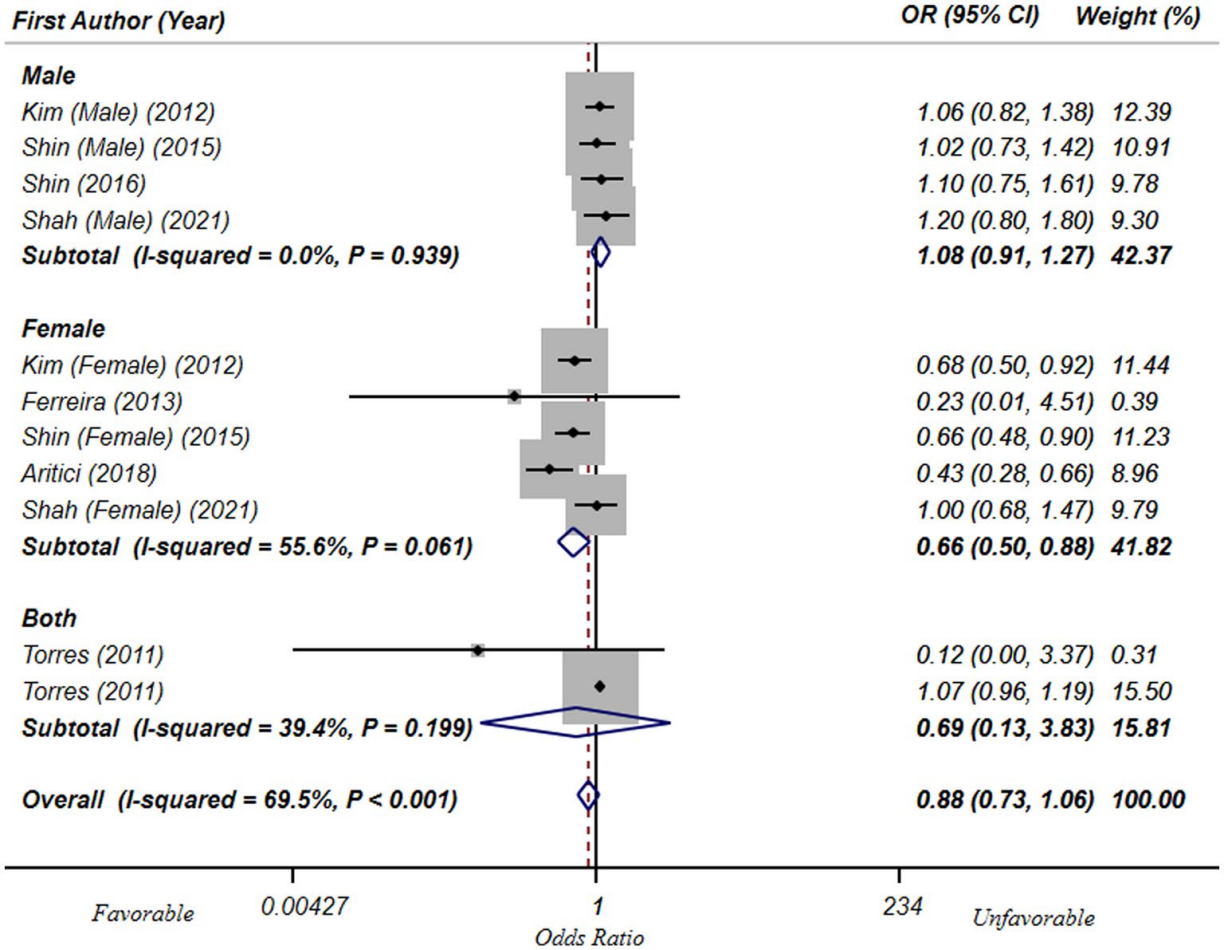
Forest plots of the association of highest vs. lowest level of dietary Ca intake and T2DM/ hyperglycemia in cross-sectional, stratified by sex.

Meta-analysis on 8 cross-sectional studies^15,16,19,20,22,24,25,30^ (including 40,614 participants) represented no significant relationship between dietary Ca intake and odds of T2DM/hyperglycemia (OR: 0.88; 95% CI: 0.73, 1.06) (Fig. 5). Considering the significant between-study heterogeneity (I^2^ = 69.5, P_Q-test_ < 0.001), subgroup analysis was performed based on gender. Although the heterogeneity was eliminated in males (I^2^ = 0.0, P_Q-test_ = 0.93) and both gender together (I^2^ = 39.4, P_Q-test_ = 0.19), there was no significant association in these subgroups [(OR: 1.08; 95% CI: 0.91, 1.27), (OR: 0.69; 95% CI: 0.13, 3.83), respectively]. However, in females, higher Ca intake was significantly related to a 34% lower odds of T2DM/hyperglycemia (OR: 0.66; 95% CI: 0.50, 0.88), without significant heterogeneity in this subgroup (I^2^ = 55.6, P_Q-test_ = 0.06) (Fig. 5). Subgroup analyses based on other confounders were done and the results are shown in Table 4. In all of these subgroups, no significant association was found, except in one study that used food record questionnaire to assess dietary intakes. Meta-regression showed that mean age of participants (β = 0.017, P = 0.25, I^2^_residual_ = 68.1%), latitude (β =  − 0.015, P = 0.52, I^2^_residual_ = 64.33%), longitude (β = 0.002, P = 0.39, I^2^_residual_ = 71.36%) and the quality of included studies (β = 0.086, P = 0.49, I^2^_residual_ = 71.69%) could not explain the observed heterogeneity. Based on sensitivity analysis, no particular study significantly affected the overall estimate. The visual inspection of the funnel plot, Begg’s test (P = 0.24) and Egger’s test (P = 0.09) showed no significant publication bias.

**Table 4:** Results of subgroup-analyses of dietary Ca intake in relation to T2DM/hyperglycemia in cross-sectional studies.

	No. of effect sizes	OR (95% CI)	P within^1^	I^2^ (%)	P between^2^
Overall	11	0.88 (0.73, 1.06)	0.00	69.5	
Representativeness of population				0.25	
Representative	7	0.93 (0.77, 1.11)	0.05	51.0	
Non-representative	4	0.59 (0.25, 1.35)	< 0.001	84.4	
Questionnaire					0.00
FFQ	7	0.92 (0.76, 1.10)	0.01	61.9	
Recall	3	1.07 (0.81, 1.43)	0.35	3.7	
Record	**1**	**0.43 (0.28, 0.66)**	-	-	
Outcome					0.45
T2D	3	1.07 ( 0.81, 1.43)	0.35	3.7	
High blood glucose (HBG)	8	0.83 (0.66, 1.04)	< 0.001	76.8	
Health status of participants				0.00	
Healthy	8	0.82 (0.64, 1.04)	0.00	69.0	
Un healthy	3	1.07 (0.96, 1.19)	0.43	0.0	
Mean age					0.28
< 50	6	0.82 (0.53, 1.27)	0.00	70.9	
≥ 50	5	0.90 (0.73, 1.11)	0.00	72.5	
Asian vs. Non-Asian counties				0.00	
Asian	6	0.79 (0.61, 1.04)	0.00	74.8	
Non-Asian	5	1.07 (0.97, 1.18)	0.54	0.0	
Development status of study location			0.01		
Developed	8	0.86 (0.68, 1.07)	0.00	70.3	
Developing	3	0.71 (0.23, 2.21)	0.26	25.1	
Quality score^3^					0.23
Low quality (Scores ≤ 7)	3	0.62 (0.26, 1.50)	< 0.001	89.1	
High quality (Scores > 7)	8	0.92 (0.77, 1.10)	0.07	46.4	

^1^P for heterogeneity, within subgroup.

^2^P for heterogeneity, between subgroups.

^3^Quality Scores were according to Newcastle–Ottawa Scale.

### Findings from dose–response meta-analysis of dietary Ca intake and T2DM/hyperglycemia in cross-sectional studies

We conducted a linear dose–response analysis on 8 cross-sectional studies with sufficient data^15,16,19,22–25,30^ (including 43,853 total participants with 8,786 cases of T2DM/hyperglycemia). There was no significant linear relation between an increase of 300, or 600, or 1000 mg/day dietary Ca intake and odds of T2DM/hyperglycemia [(OR: 0.99; 95% CI: 0.96, 1.03); (OR: 0.98; 95% CI: 0.92, 1.06); and (OR: 0.98; 95% CI: 0.87, 1.10), respectively) (Supplemental Fig. 3, 4 and 5). However, non-linear dose–response analysis on 4 cross-sectional studies with 3 or more dietary Ca levels^15,16,19,30^ (including 40,261 participants with 8,214 cases of T2DM/hyperglycemia) revealed a significant relation (P_non-linearity_ = 0.02) (Supplemental Fig. 6).

### Quality of the evidence

GRADE evidence profile for dietary Ca intake in relation to T2D and hyperglycemia is presented in Supplemental Table 5. The certainty of evidence was rated as "high quality" for cohort studies and "moderate quality" for cross-sectional investigations. The endpoint for both cohort and cross-sectional studies was upgraded for "risk of bias", "inconsistency" and "indirectness". For both cohort and cross-sectional studies, 95% CI of overall effect contained a minimal value of 0.75; so, the certainty of evidence was downgraded for "imprecision". Cohort studies had also reported essential data for dose–response analysis; so, the endpoint of these investigations was upgraded for “other considerations". Cross-sectional studies did not provide enough data for dose–response analysis and the endpoint for these studies was downgraded for “other considerations".

## Discussion

This meta-analysis documented that in prospective cohort studies highest category of Ca intake, compared to lowest one, was significantly related to decreased risk of T2DM in adults. This inverse significant relation between dietary Ca intake and T2DM was also confirmed in almost all subgroups. Moreover, based on linear dose–response analysis, each 300, 600 and 1000 (mg/day) increment in dietary Ca intake was respectively associated with 7, 14 and 23% reduced risk of T2DM. It seems that the threshold effect of dietary Ca intake was around 750 mg/day; a steeper reduction in risk of T2DM was observed when dietary Ca intake increased from low levels to 750 mg/day. There was no significant relation between dietary Ca intake above 750 mg/day and risk of T2DM. Nevertheless, meta-analysis on cross-sectional studies revealed an inverse significant association between dietary Ca intake and T2DM/hyperglycemia only in the female population and there was no significant linear relationship in cross-sectional studies.

Previous investigations documented straight associations between T2DM and risk of chronic conditions including hypertension, dyslipidemia, obesity and degenerative diseases such as Parkinson's disease^40,41^. In addition, T2DM is correlated to double risk of mortality ^42,43^; so, management the increasing rate of T2DM prevalence is a major issue. In the current analysis, we illustrated that subjects with lower dietary Ca intake have higher risk of T2DM; this point could be clinically recommended to individuals to increase their Ca intake via food in a hope to decrease the risk of hyperglycemia and subsequently T2DM.

In line with our study, previous systematic reviews and meta-analyses of epidemiologic studies investigated the relation of Ca intake with the risk of non-communicable disease (NCDs). These researches revealed inverse significant relations between Ca intake and colorectal cancer^44^, breast cancer^45^, ovarian cancer^46^ and stroke^47^. Nevertheless, prior investigations could not confirm significant relation between Ca intake and CVD^48^ and depression^49^. Yang et al.^8^ conducted a meta-analysis on prospective cohort studies and randomized clinical trials (RCTs). They found that dietary Ca intake ranging from 200 to 1500 mg/day did not affect the risk of CVD, coronary heart disease (CHD), or stroke. However, Ca supplements could raise the risk of CHD, and especially myocardial infarction (MI). In 2012, Dong et al.^26^ have conducted a meta-analysis on prospective studies and documented an inverse significant relation between Ca intake and T2DM; however, they reported that this relation was dependent on magnesium intake and the overall estimate was not significant in studies that controlled the effect of magnesium intake. In contrast, our analysis illustrated an inverse significant relationship between dietary Ca intake and T2DM in the subgroup of studies that made adjustment for magnesium intake.

In the current analysis, an inverse significant relationship between dietary Ca intake and T2DM was observed in cohort studies. In addition, a significant linear relation was documented. Notably, the threshold for the beneficial effect of dietary Ca intake was around 750 mg/day. Similar to our study, Wang et al.^50^ conducted a meta-analysis on prospective cohort studies and documented a U-shaped relation between Ca intake and CVD mortality; such that, there was significant relation between dietary Ca intake lower or higher than around 800 mg/day and increased risk of mortality from CVD. For all-cause mortality, they found a threshold effect at Ca intakes around 900 mg/day and the risk did not decrease further, at intakes more than 900 mg/day. They have also found a threshold effect of 900 mg/day for all-cause mortality; the risk did not decline at Ca intakes more than 900 mg/day.

Although we documented that Ca intake was related to a lower risk of T2DM in prospective cohort studies, no significant relationship was found in the whole population in cross-sectional studies. In this case, some points might affect the results and should be considered. First, in cross-sectional studies, the number of effect sizes and the sample size of studies were lower than those in cohort studies (n = 40,614 vs. 255,744). Second, all cohort studies were conducted on apparently healthy individuals who were representative of the whole adult populations; so, the results of these cohorts could be generalizable to the whole population. However, both representative and non-representative populations were included in cross-sectional studies. A small number of eligible cross-sectional studies (n = 2) were also conducted on patients. Third, most cohort studies controlled the effect of Mg intake; while none of the cross-sectional studies made this adjustment. It is also should be considered that all eligible cohorts had used FFQs to measure dietary Ca intake; whereas cross-sectional studies have applied FFQs, food record and food recall questionnaires. Several replications of 24-h dietary recalls or records (at least 3 to 5) are needed to estimate the usual dietary intakes.

Our stratified analysis by gender among cohort studies revealed an inverse association between highest vs. lowest Ca intake and risk of T2DM in females and both genders together, but the relation was not significant in males. Similarly, in cross-sectional studies, higher Ca intake was related to the reduced odds of T2DM/hyperglycemia in females. Increasing Ca absorption and its bioavailability in premenopausal women by estrogen hormone might be the reason for different findings in men and women^51^.

Although previous studies have documented significant relation between dietary Ca intake and T2DM, they could not exactly clarify underlying mechanisms. Some pathways were suggested to explain this relationship. Earlier investigations documented that hypocalcemia due to vitamin D deficiency could lead to insulin resistance and impaired insulin secretion^52^. An experimental study showed that Ca intake could increase the extracellular Ca, affect the beta cells of pancreas, and improve insulin secretion and insulin resistance in rats with vitamin D deficiency, while vitamin D supplementation had no significant effect^53^. In human studies, hypocalcemia in non-diabetic individuals could also impair insulin release^54,55^, whereas vitamin D supplementation had no beneficial effect on glucose/insulin homeostasis^56,57^ and the impact of Ca intake was not tested in this situation. Furthermore, oral calcium load could lead to increased glucose-induced insulin secretion in patients with diabetes^58^. In addition, dietary Ca intake is related to reduced lipogenesis, less inflammation and increased lipolysis. Therefore, it seems that higher dietary Ca intake would decrease obesity, inflammation and consequently insulin resistance and T2DM^22,59^. Furthermore, there are mechanisms for explanation the cellular role of Ca including, first, insulin secretion is calcium-dependent, any change in Ca flux might have an adverse effect on this secretion^60^; second, Ca plays an important role in insulin action. The phosphorylation of insulin receptors depends on Ca^61^. Hypocalcemia could impair insulin signal transduction^62,63^ and decrease the activity of glucose transporter-4^63,64^. Therefore, in insulin-target tissues (such as skeletal muscle and adipose tissue) Ca is necessary for insulin-mediated intracellular processes^62,65^.

As far as we know, this is the first meta-analysis that examined a dose–response relationship between dietary Ca intake and the risk of T2DM/hyperglycemia in epidemiologic studies. In the current analysis, a large population of adults was included and we found significant relation in cohort studies, in a dose–response fashion. Considering the representative population of cohort studies, the results could be generalized to the whole adult population. Additionally, we performed subgroup analyses based on several confounders. GRADE approach provided the certainty that dietary Ca intake is related to reduced odds of T2DM and may have a role in decreasing hyperglycemia. However, some limitations should be kept in mind. First, only a few included studies have separately reported the sources of Ca intake (dietary, supplemental, plant-based and animal-based); therefore, we could only consider dietary Ca intake as the exposure. Second, none of the included cross-sectional studies controlled the impact of Mg intake. Third, cross-sectional studies have used different questionnaires to examine dietary Ca intake including FFQ, food record and food recall. Fourth, included studies had a wide range of follow-up, centers of data collection and populations. Additionally, participants had a wide range of dietary calcium intake due to the various dietary intakes in each region. Fifth, some included studies had significant sources of bias because of having low sample size, non-representative study population, and high rate of dropout or using invalid food questionnaire. Finally, gray literatures that have not usually passed peer review process and might be low quality or significant sources of bias, were not included in the current analysis. The above-mentioned restrictions might lead to insignificant results and moderate between-study heterogeneity in cross-sectional studies. Furthermore, only a few studies investigated the relation of dietary Ca intake with abnormal glucose homeostasis and their results were inconsistent. Therefore, there was low evidence to support the finding in this field.

In conclusion, this meta-analysis illustrated an inverse association between dietary Ca intake and risk of T2DM in general adult populations in prospective cohort studies, in a dose–response manner. It seems that increasing dietary Ca intake from low levels to around 750 mg/day was related to a lower risk of T2DM. Meta-analysis on cross-sectional studies showed an inverse relation between dietary Ca intake and T2DM/hyperglycemia only in females.

## Supplementary Information

Supplementary Information.

## Abbreviations

OR: Odds ratio
RR: Relative risks
95% CI: 95% Confidence intervals; HR, Hazard ratio;
β: Standard regression coefficient
B: Un-standard regression coefficient
T2DM: Type 2 diabetes mellitus
CVDs: Cardiovascular disease
PRISMA: Preferred Reporting Items for Systematic Reviews and Meta-Analyses guideline
NOS: Newcastle–Ottawa scale
GDM: Gestational diabetes mellitus
T1DM: Type 1 diabetes mellitus
GRADE: Grading of Recommendations
NCD: Non-communicable disease
BMI: Body mass index
MI: Myocardial infarction
MR Cohort: Multi-Rural Communities Cohort
KoGES: Korean Genome and Epidemiology Study
FFQ: Food frequency questionnaire
HBG: High blood glucose
CHD: Coronary heart disease
NHANES: National health and nutrition examination surveys

## References

[1] Shaw JE, Sicree RA, Zimmet PZ (2010). Global estimates of the prevalence of diabetes for 2010 and 2030. Diabetes Res. Clin. Pract..

[2] Baynes HW (2015). Classification, pathophysiology, diagnosis and management of diabetes mellitus. J. Diabetes Metab..

[3] Eppens MC, Craig ME, Cusumano J, Hing S, Chan AK, Howard NJ, Silink M, Donaghue KC (2006). Prevalence of diabetes complications in adolescents with type 2 compared with type 1 diabetes. J. Diabetes Med..

[4] Kannel WB, McGee DLJJ (1979). Diabetes and cardiovascular disease: the Framingham study..

[5] Manson JE, Colditz GA, Stampfer MJ, Willett WC, Krolewski AS, Rosner B, Arky RA, Speizer FE, Hennekens CH (1991). A prospective study of maturity-onset diabetes mellitus and risk of coronary heart disease and stroke in women. Arch. Intern. Med..

[6] Shaw JE, Sicree RA, Zimmet PZ (2010). practice c: **Global estimates of the prevalence of diabetes for 2010 and 2030**. J. Diabetics Res..

[7] Larsson SC, Orsini N, Wolk A (2013). Dietary calcium intake and risk of stroke: a dose-response meta-analysis. Am. Clin. Nutr..

[8] Yang C, Shi X, Xia H, Yang X, Liu H, Pan D, Sun G (2020). The evidence and controversy between dietary calcium intake and calcium supplementation and the risk of cardiovascular disease: a systematic review and meta-analysis of cohort studies and randomized controlled trials. J. Am. Coll. Nutr..

[9] Ahn J, Albanes D, Peters U, Schatzkin A, Lim U, Freedman M, Chatterjee N, Andriole GL, Leitzmann MF, Hayes RB (2007). Dairy products, calcium intake, and risk of prostate cancer in the prostate, lung, colorectal, and ovarian cancer screening trial. Cancer Epidemiol. Prevent. Biomark..

[10] Goldberg P (1974). Multiple sclerosis: vitamin D and calcium as environmental determinants of prevalence: (A viewpoint) part 2. Biochemical and genetic factors. Int. J. Environ. Stud..

[11] Pittas AG, Lau J, Hu FB, Dawson-Hughes B (2007). The role of vitamin D and calcium in type 2 diabetes. A systematic review and meta-analysis. J. Clin. Endocrinol. Metabol..

[12] Heshmati J, Sepidarkish M, Namazi N, Shokri F, Yavari M, Fazelian S, Khorshidi M, Shidfar F (2019). Impact of dietary calcium supplement on circulating lipoprotein concentrations and atherogenic indices in overweight and obese individuals: A systematic review. J. Dietary Suppl..

[13] Talaei M, Pan A, Yuan J-M, Koh W-P (2018). Dairy intake and risk of type 2 diabetes. Clin. Nutr..

[14] Villegas R, Gao Y-T, Dai Q, Yang G, Cai H, Li H, Zheng W, Shu XO (2009). Dietary calcium and magnesium intakes and the risk of type 2 diabetes: the Shanghai Women’s Health Study. Am. J. Clin. Nutr..

[15] Kim K, Yang YJ, Kim K, Kim MK (2012). Interactions of single nucleotide polymorphisms with dietary calcium intake on the risk of metabolic syndrome. Am. J. Clin. Nutr..

[16] Shin SK, Kim MK, Lee Y-H, Shin DH, Shin M-H, Chun B-Y, Choi BY (2015). The cross-sectional relationship between dietary calcium intake and metabolic syndrome among men and women aged 40 or older in rural areas of Korea. Nutr. Res. Pract..

[17] Gagnon C, Lu ZX, Magliano DJ, Dunstan DW, Shaw JE, Zimmet PZ, Sikaris K, Grantham N, Ebeling PR, Daly RM (2011). Serum 25-hydroxyvitamin D, calcium intake, and risk of type 2 diabetes after 5 years: Results from a national, population-based prospective study (the Australian Diabetes, Obesity and Lifestyle study). Diabetes Care.

[18] Oh J, Woo H, Kim M, Lee Y-H, Shin D, Shin M-H, Choi B (2017). Dietary total, animal, vegetable calcium and type 2 diabetes incidence among Korean adults: The Korean Multi-Rural Communities Cohort (MRCohort). Nutr. Metab. Cardiovasc. Dis..

[19] Shah IU, Sameen A, Manzoor MF, Ahmed Z, Gao J, Farooq U, Siddiqi SM, Siddique R, Habib A, Sun C (2021). Association of dietary calcium, magnesium, and vitamin D with type 2 diabetes among US adults: National health and nutrition examination survey 2007–2014—A cross-sectional study. Food Sci. Nutr..

[20] Aritici, G. & Baş, M. Metabolic syndrome and calcium: The effects on body composition and biochemical parameters among premenopausal women. (2018).

[21] Beydoun MA, Fanelli-Kuczmarski MT, Beydoun HA, Dore GA, Canas JA, Evans MK, Zonderman AB (2018). Dairy product consumption and its association with metabolic disturbance in a prospective study of urban adults. Br. J. Nutr..

[22] da Silva FT, Torres MRSG, Sanjuliani AF (2013). Dietary calcium intake is associated with adiposity, metabolic profile, inflammatory state and blood pressure, but not with erythrocyte intracellular calcium and endothelial function in healthy pre-menopausal women. Br. J. Nutr..

[23] Pannu PK, Soares MJ, Piers LS, Zhao Y, Ansari Z (2017). The association of vitamin D status and dietary calcium intake with individual components of the metabolic syndrome: a population-based study in Victoria, Australia. Cardiovasc. Endocrinol..

[24] Torres MRSG, da Silva FT, Carvalho DC, Sanjuliani AF (2011). Dietary calcium intake and its relationship with adiposity and metabolic profile in hypertensive patients. Nutrition.

[25] Torres MRSG, Gioseffi C, Cardoso LG, Barroso SG, Sanjuliani AF, Souza E (2011). A Pilot study on the relation between dietary calcium and clinical parameters in renal transplant recipients. J. Ren. Nutr..

[26] Dong J, Qin L (2012). Dietary calcium intake and risk of type 2 diabetes: possible confounding by magnesium. Eur. J. Clin. Nutr..

[27] Wu F, Juonala M, Pahkala K, Buscot M-J, Sabin MA, Pitkänen N, Rönnemaa T, Jula A, Lehtimäki T, Hutri-Kähönen N (2019). Youth and long-term dietary calcium intake with risk of impaired glucose metabolism and type 2 diabetes in adulthood. J. Clin. Endocrinol. Metab..

[28] Woo HW, Lim Y-H, Kim MK, Shin J, Lee Y-H, Shin DH, Shin M-H, Choi BY (2020). Prospective associations between total, animal, and vegetable calcium intake and metabolic syndrome in adults aged 40 years and older. Clin. Nutr..

[29] Kim K-N, Oh S-Y, Hong Y-C (2018). Associations of serum calcium levels and dietary calcium intake with incident type 2 diabetes over 10 years: the Korean Genome and Epidemiology Study (KoGES). Diabetol. Metab. Syndr..

[30] Shin B-R, Choi Y-K, Kim H-N, Song S-W (2016). High dietary calcium intake and a lack of dairy consumption are associated with metabolic syndrome in obese males: the Korean National Health and Nutrition Examination Survey 2010 to 2012. Nutr. Res..

[31] Moore-Schiltz L, Albert JM, Singer ME, Swain J, Nock NL (2015). Dietary intake of calcium and magnesium and the metabolic syndrome in the National Health and Nutrition Examination (NHANES) 2001–2010 data. Br. J. Nutr..

[32] Wells, G. A., Tugwell, P., O’Connell, D., Welch, V., Peterson, J., Shea, B. & Losos, M. The Newcastle-Ottawa Scale (NOS) for assessing the quality of nonrandomized studies in meta-analyses. (2015).

[33] Guyatt G, Oxman AD, Akl EA, Kunz R, Vist G, Brozek J, Norris S, Falck-Ytter Y, Glasziou P, DeBeer H (2011). GRADE guidelines: 1. Introduction—GRADE evidence profiles and summary of findings tables. J. Clin. Epidemiol..

[34] GRADEpro GDT. GRADEpro Guideline Development Tool [Software]. (Developed by Evidence Prime, Inc.): McMaster University; 2015. http://www.gradepro.org. Accessed 12 Nov 2021.

[35] Greenland S, Longnecker MP (1992). Methods for trend estimation from summarized dose-response data, with applications to meta-analysis. Am. J. Epidemiol..

[36] Orsini N, Bellocco R, Greenland S (2006). Generalized least squares for trend estimation of summarized dose–response data. Stand. Genomic Sci..

[37] van Dam RM, Hu FB, Rosenberg L, Krishnan S, Palmer JR (2006). Dietary calcium and magnesium, major food sources, and risk of type 2 diabetes in US black women. Diabetes Care.

[38] Pittas AG, Dawson-Hughes B, Li T, Van Dam RM, Willett WC, Manson JE, Hu FB (2006). Vitamin D and calcium intake in relation to type 2 diabetes in women. Diabetes Care.

[39] Kirii K, Mizoue T, Iso H, Takahashi Y, Kato M, Inoue M, Noda M, Tsugane S (2009). Calcium, vitamin D and dairy intake in relation to type 2 diabetes risk in a Japanese cohort. Diabetologia.

[40] Xu Q, Park Y, Huang X, Hollenbeck A, Blair A, Schatzkin A, Chen H (2011). Diabetes and risk of Parkinson’s disease. Diabetes Care.

[41] Moon JH, Roh E, Oh TJ, Kim KM, Moon JH, Lim S, Jang HC, Choi SH (2017). Increased risk of metabolic disorders in healthy young adults with family history of diabetes: from the Korea National Health and Nutrition Survey. Diabetol. Metab. Syndr..

[42] Group NDD, Diabetes NIo, Digestive, Diseases K: *Diabetes in America.* National Institutes of Health, National Institute of Diabetes and Digestive (1995).

[43] Tancredi M, Rosengren A, Svensson A-M, Kosiborod M, Pivodic A, Gudbjörnsdottir S, Wedel H, Clements M, Dahlqvist S, Lind M (2015). Excess mortality among persons with type 2 diabetes. N. Engl. J. Med..

[44] Keum N, Lee DH, Greenwood DC, Zhang X, Giovannucci EL (2015). Calcium intake and colorectal adenoma risk: Dose-response meta-analysis of prospective observational studies. Int. J. Cancer.

[45] Hidayat K, Chen G-C, Zhang R, Du X, Zou S-Y, Shi B-M, Qin L-Q (2016). Calcium intake and breast cancer risk: Meta-analysis of prospective cohort studies. Br. J. Nutr..

[46] Song X, Li Z, Ji X, Zhang D (2017). Calcium intake and the risk of ovarian cancer: a meta-analysis. Nutrients.

[47] Tian D-y (2015). Tian, J., Shi, C.-h, Song, B., Wu, J., Ji, Y., Wang, R.-h, Mao, C.-y, Sun, S.-l. & Xu, Y.-m. Calcium intake and the risk of stroke: An up-dated meta-analysis of prospective studies. Asia Pac. J. Clin. Nutr..

[48] Chung M, Tang AM, Fu Z, Wang DD, Newberry SJ (2016). Calcium intake and cardiovascular disease risk: An updated systematic review and meta-analysis. Ann. Intern. Med..

[49] Li B, Lv J, Wang W, Zhang D (2017). Dietary magnesium and calcium intake and risk of depression in the general population: A meta-analysis. Aust. N. Z. J. Psychiatry.

[50] Wang X, Chen H, Ouyang Y, Liu J, Zhao G, Bao W, Yan M (2014). Dietary calcium intake and mortality risk from cardiovascular disease and all causes: a meta-analysis of prospective cohort studies. BMC Med..

[51] Heaney RP, Recker RR, Stegman MR, Moy AJ (1989). Calcium absorption in women: Relationships to calcium intake, estrogen status, and age. J. Bone Miner. Res..

[52] Alvarez JA, Ashraf A (2010). Role of vitamin D in insulin secretion and insulin sensitivity for glucose homeostasis. Int. J. Endocrinol..

[53] Ismail A, Namala R (2000). Impaired glucose tolerance in vitamin D deficiency can be corrected by calcium. J. Nutr. Biochem..

[54] Yasuda K, Hurukawa Y, Okuyama M, Kikuchi M, Yoshinaga K (1975). Glucose tolerance and insulin secretion in patients with parathyroid disorders: Effect of serum calcium on insulin release. N. Engl. J. Med..

[55] Gedik O, Zileli M (1977). Effects of hypocalcemia and theophylline on glucose tolerance and insulin release in human beings. Diabetes.

[56] Tai K, Need AG, Horowitz M, Chapman IM (2008). Glucose tolerance and vitamin D: Effects of treating vitamin D deficiency. Nutrition.

[57] Lind L, Pollare T, Hvarfner A, Lithell H, Sørensen O, Ljunghall S (1989). Long-term treatment with active vitamin D (alphacalcidol) in middle-aged men with impaired glucose tolerance. Effects on insulin secretion and sensitivity, glucose tolerance and blood pressure. Diabetes Res. (Edinburgh, Scotland).

[58] Fujita T (1978). Sakagami, Y., Tomita, T., Okamoto, Y. & Oku, H. Insulin secretion after oral calcium load. Endocrinol. Jpn..

[59] Shi H, DiRienzo D, Zemel MB (2001). Effects of dietary calcium on adipocyte lipid metabolism and body weight regulation in energy-restricted aP2-agouti transgenic mice. FASEB J..

[60] Milner R, Hales C (1967). The role of calcium and magnesium in insulin secretion from rabbit pancreas studied in vitro. Diabetologia.

[61] Plehwe WE, Williams PF, Caterson ID, Harrison LC, Turtle J (1983). Calcium-dependence of insulin receptor phosphorylation. Biochem. J..

[62] Williams P, Caterson I, Cooney G, Zilkens R, Turtle J (1990). High affinity insulin binding and insulin receptor-effector coupling: modulation by Ca2+. Cell Calcium.

[63] Zemel MB (1998). Nutritional and endocrine modulation of intracellular calcium: implications in obesity, insulin resistance and hypertension. Mol. Cell. Effects Nutr.Disease Process..

[64] Reusch JE-B, Begum N, Sussman KE, Draznin B (1991). Regulation of GLUT-4 phosphorylation by intracellular calcium in adipocytes. Endocrinology.

[65] Ojuka EO (2004). Role of calcium and AMP kinase in the regulation of mitochondrial biogenesis and GLUT4 levels in muscle. Proc. Nutr. Soc..

