# Supplementary material for: Dietary calcium intake in relation to type-2 diabetes and hyperglycemia in adults: A systematic review and dose–response meta-analysis of epidemiologic studies
Source: Sci Rep. 2022 Jan 20;12:1050. doi: 10.1038/s41598-022-05144-8 (PMC8776796; doi:10.1038/s41598-022-05144-8)
Supplement: Supplementary file 1 — Supplementary Information. [file 41598_2022_5144_MOESM1_ESM.pdf]

Legend to Supplemental Figures and Tables:

Supplemental Figure 1. *Forest plots of linear dose-response meta-analysis of the association between each 600 mg/day increment in dietary Ca intake levels and T2DM in prospective cohort studies with representative adult populations.*

Supplemental Figure 2. *Forest plots of linear dose-response meta-analysis of the association between each 1000 mg/day increment in dietary Ca intake levels and T2DM in prospective cohort studies with representative adult populations.*

Supplemental Figure 3. *Forest plots of linear dose-response meta-analysis of the association between each 300 mg/day increment in dietary Ca intake levels and T2DM/ hyperglycemia in cross-sectional studies.*

Supplemental Figure 4. *Forest plots of linear dose-response meta-analysis of the association between each 600 mg/day increment in dietary Ca intake levels and T2DM/ hyperglycemia in cross-sectional studies.*

Supplemental Figure 5. *Forest plots of linear dose-response meta-analysis of the association between each 1000 mg/day increment in dietary Ca intake levels and T2DM/ hyperglycemia in cross-sectional studies.*

Supplemental Figure 6. *Non-linear dose–response association between dietary Ca intake levels and T2DM/ hyperglycemia in cross-sectional studies. - - - , Linear model;* **____**, *spline model.*

Supplemental Table 1. *MeSH and non-MeSH terms that were used in the systematic search*

Supplemental Table 2. *PRISMA checklist*

Supplemental Table 3*. Details of more relevant studies that were excluded in the first step of the screening.*

Supplemental Table 4*. Details of quality assessment of eligible studies.*

Supplementary Table 5. *GRADE evidence profile for dietary Ca intake in relation to T2D and hyperglycemia*

Supplemental Table 1. *MeSH and non-MeSH terms that were used in the systematic search*

Database	***Syntax***	***Results***
PubMed	*(("Calcium intake"[Title/Abstract] OR "Dietary Calcium intake" [Title/Abstract] OR "Dietary Calcium")[Title/Abstract])) AND ((diabetes[Title/Abstract] OR prediabetes[Title/Abstract] OR "insulin resistance"[Title/Abstract] OR HOMA-IR[Title/Abstract] OR glucose[Title/Abstract] OR insulin[Title/Abstract] OR FBS[Title/Abstract] OR FBG[Title/Abstract] OR FPS[Title/Abstract] OR FPG[Title/Abstract] OR "Glycemic control"[Title/Abstract] OR "HbA1c"[Title/Abstract] OR "Glycosilated Hemoglobin"[Title/Abstract] OR "IFG"[Title/Abstract] OR "insulin sensitivity"[Title/Abstract] OR "homeostasis model assessment"[Title/Abstract] OR "Glucose Tolerance Test"[Title/Abstract] OR "Insulin"[Mesh] OR "Glucose"[Mesh] OR "Prediabetic State"[Mesh] OR "Diabetes Mellitus"[Mesh] OR "Diabetes Mellitus, Type 2"[Mesh]))*	*806*
Web Of Sciences	*(“Calcium intake” OR “Dietary Calcium intake” OR “Dietary Calcium”) AND (diabetes OR prediabetes OR "insulin resistance" OR “HOMA-IR” OR glucose OR insulin OR FBS OR FBG OR FPS OR FPG OR "Glycemic control" OR "HbA1c" OR "Glycosilated Hemoglobin" OR "IFG" OR "insulin sensitivity" OR "homeostasis model assessment" OR "Glucose Tolerance Test" OR "Insulin" OR "Glucose" OR "Prediabetic State" OR "Diabetes Mellitus" OR "Diabetes Mellitus, Type 2")*	*796*
Scopus	*(TITLE-ABS-KEY (("Calcium intake" OR "Dietary Calcium intake" OR "Dietary Calcium")) AND TITLE-ABS-KEY ((diabetes OR prediabetes OR "insulin resistance" OR "HOMA-IR" OR glucose OR insulin OR fbs OR fbg OR fps OR fpg OR "Glycemic control" OR "HbA1c" OR "Glycosilated Hemoglobin" OR "IFG" OR "insulin sensitivity" OR "homeostasis model assessment" OR "Glucose Tolerance Test" OR "Insulin" OR "Glucose" OR "Prediabetic State" OR "Diabetes Mellitus" OR "Diabetes Mellitus, Type 2")))*	*1470*

Embase	*('Calcium intake' :ti,ab,kw OR 'Dietary Calcium intake':ti,ab,kw OR 'Dietary Calcium':ti,ab,kw) AND (diabetes:ti,ab,kw OR 'Diabetes Mellitus':ti,ab,kw OR 'Diabetes Mellitus, Type 2':ti,ab,kw OR prediabetes:ti,ab,kw OR 'Prediabetic State':ti,ab,kw OR 'insulin resistance':ti,ab,kw OR 'homeostasis model assessment':ti,ab,kw OR HOMA-IR:ti,ab,kw OR glucose:ti,ab,kw OR insulin:ti,ab,kw OR FBS :ti,ab,kw OR FBG:ti,ab,kw OR FPS OR FPG OR 'Glycemic control' OR HbA1c OR "Glycosylated Hemoglobin" OR IFG OR "insulin sensitivity" OR "Glucose Tolerance Test")*	*852*

Supplemental Table 2. *PRISMA Checklist*

Section/topic	#	Checklist item	Reported on page #
**TITLE**			
Title	1	Identify the report as a systematic review, meta-analysis, or both.	1
**ABSTRACT**			
Structured summary	2	Provide a structured summary including, as applicable: background; objectives; data sources; study eligibility criteria, participants, and interventions; study appraisal and synthesis methods; results; limitations; conclusions and implications of key findings; systematic review registration number.	3,4
**INTRODUCTION**			
Rationale	3	Describe the rationale for the review in the context of what is already known.	5-6
Objectives	4	Provide an explicit statement of questions being addressed with reference to participants, interventions, comparisons, outcomes, and study design (PICOS).	5-6, Table 1
**METHODS**			
Protocol and registration	5	Indicate if a review protocol exists, if and where it can be accessed (e.g., Web address), and, if available, provide registration information including registration number.	7
Eligibility criteria	6	Specify study characteristics (e.g., PICOS, length of follow-up) and report characteristics (e.g., years considered, language, publication status) used as criteria for eligibility, giving rationale.	6-8
Information sources	7	Describe all information sources (e.g., databases with dates of coverage, contact with study authors to identify additional studies) in the search and date last searched.	6-7
Search	8	Present full electronic search strategy for at least one database, including any limits used, such that it could be repeated.	6-7
Study selection	9	State the process for selecting studies (i.e., screening, eligibility, included in systematic review, and, if applicable, included in the meta-analysis).	6-8
Data collection process	10	Describe method of data extraction from reports (e.g., piloted forms, independently, in duplicate) and any processes for obtaining and confirming data from investigators.	8
Data items	11	List and define all variables for which data were sought (e.g., PICOS, funding sources) and any assumptions and simplifications made.	6-8, Table 1
Risk of bias in individual studies	12	Describe methods used for assessing risk of bias of individual studies (including specification of whether this was done at the study or outcome level), and how this information is to be used in any data synthesis.	8-9
Summary measures	13	State the principal summary measures (e.g., risk ratio, difference in means).	9,10
Synthesis of results	14	Describe the methods of handling data and combining results of studies, if done, including measures of consistency (e.g., I^2) for each meta-analysis.	9,10
Risk of bias across studies	15	Specify any assessment of risk of bias that may affect the cumulative evidence (e.g., publication bias, selective reporting within studies).	9,10

Additional analyses	*16*	*Describe methods of additional analyses (e.g., sensitivity or subgroup analyses, meta-regression), if done, indicating which were pre-specified.*	*9,10*
RESULTS			
Study selection	*17*	*Give numbers of studies screened, assessed for eligibility, and included in the review, with reasons for exclusions at each stage, ideally with a flow diagram.*	*11, Figure 1, Supplemental Table 3*
Study characteristics	*18*	*For each study, present characteristics for which data were extracted (e.g., study size, PICOS, follow-up period) and provide the citations.*	*11-12, Table 2*
Risk of bias within studies	*19*	*Present data on risk of bias of each study and, if available, any outcome level assessment (see item 12).*	*12-15*
Results of individual studies	*20*	*For all outcomes considered (benefits or harms), present, for each study: (a) simple summary data for each intervention group (b) effect estimates and confidence intervals, ideally with a forest plot.*	*12-15*
Synthesis of results	*21*	*Present results of each meta-analysis done, including confidence intervals and measures of consistency.*	*12-15*
Risk of bias across studies	*22*	*Present results of any assessment of risk of bias across studies (see Item 15).*	*12-15*
Additional analysis	*23*	*Give results of additional analyses, if done (e.g., sensitivity or subgroup analyses, meta-regression [see Item 16]).*	*12-15*
DISCUSSION			
Summary of evidence	*24*	*Summarize the main findings including the strength of evidence for each main outcome; consider their relevance to key groups (e.g., healthcare providers, users, and policy makers).*	*15-16*
Limitations	*25*	*Discuss limitations at study and outcome level (e.g., risk of bias), and at review-level (e.g., incomplete retrieval of identified research, reporting bias).*	*19-20*
Conclusions	*26*	*Provide a general interpretation of the results in the context of other evidence, and implications for future research.*	*20*
FUNDING			
Funding	*27*	*Describe sources of funding for the systematic review and other support (e.g., supply of data); role of funders for the systematic review.*	*21*

From: Moher D, Liberati A, Tetzlaff J, Altman DG, The PRISMA Group (2009). Preferred Reporting Items for Systematic Reviews and Meta-Analyses: The PRISMA Statement. PLoS Med 6(7): e1000097. doi:10.1371/journal.pmed1000097

For more information, visit: ***www.prisma-statement.org***.

Supplemental Table 3. *Details of more relevant studies that were excluded.*

Reference number	***First Author (Year)***	***Title of paper***	***Reason of exclusion from current systematic review and meta-analysis***
1	*Wu (2019)*	*Youth and long-term dietary calcium intake with risk of impaired glucose metabolism and type 2 diabetes in adulthood*	*Considered dietary Ca intake as the outcome and type 2 diabetes as the exposure*
2	*Iwasaki 2019*	*Associations of Nutrient Patterns with the Prevalence of Metabolic Syndrome: Results from the Baseline Data of the Japan Multi-Institutional Collaborative Cohort Study*	*Considered the combined Ca, B2 and saturated fatty acids as the exposure*
3	*Benson (2010)*	*Type 1 Diabetes Mellitus and Components in DrinkingWater and Diet: A Population-Based, Case-Control Study in Prince Edward Island, Canada*	*Considered type 1 diabetes as the outcome*
4	*Liu, 2005*	*Dietary Calcium, Vitamin D, and the Prevalence of Metabolic Syndrome in Middle-Aged and Older U.S. Women*	*Did not report OR/RR/HR and 95%CI for the dietary Ca intake-T2D relation.*
5	*Palacios, 2020*	*Vitamin D, Calcium, Magnesium, and Potassium Consumption and Markers of Glucose Metabolism in the Hispanic Community Health Study/Study of Latinos*	*Reported geometric means of HOMA-IR, HbA1c and 2-hour glucose in different levels of dietary Ca intake.*
6	*Rogers, 2005*	*Cross-Sectional Associations of Diet and Insulin-Like Growth Factor Levels in 7- to 8-Year-Old Children*	*Reported correlation between Ca intake and IGF-I, IGFBP-3 and IGF-I/IGFBP-3 in children*
7	*Kong, 2017*	*Dietary calcium intake and risk of cardiovascular disease, stroke, and fracture in a population with low calcium intake*	*Did not report OR/RR/HR and 95%CI for the dietary Ca intake-T2D relation.*
8	*Samara, 2013*	*Dairy product consumption, calcium intakes, and metabolic syndrome–related factors over 5 years in the STANISLAS study*	*Reported standard regression coefficient*
9	*Takaya, 2018*	*A Calcium-Deficient Diet in Dams during Gestation Increases Insulin Resistance in Male Offspring*	*Reported mean±SD for HOMA-IR and HOMA-β*
10	*Jeon, 2019*	*Effects of Consuming Calcium-Rich Foods on the Incidence of Type 2 Diabetes Mellit*	*Considered the Calcium-rich foods as the exposure*
11	*Feng (2017)*	*Dietary calcium and serum 25OHD protect Chinese women from type 2 diabetes*	*Investigated dietary Ca intake-T2D in comparison healthy participants*
12	*Woo (2020)*	*Prospective associations between total, animal, and vegetable calcium intake and metabolic syndrome in adults aged 40 years and older*	*Duplicate report from population of another published study*
13	*Moore-Schiltz (2015)*	*Dietary intake of calcium and magnesium and the metabolic syndrome in the National Health and Nutrition Examination (NHANES) 2001–2010 data*	
14	*Kim (2018)*	*Associations of serum calcium levels and dietary calcium intake with incident type 2 diabetes over 10 years: the Korean Genome and Epidemiology Study (KoGES)*	Reported risk of T2D for one unit increase in log-transformed energy-adjusted dietary calcium intake, so the results could not be included in analysis.
15	*Fumeron (2011)*	*Dairy Consumption and the Incidence of Hyperglycemia and theMetabolic Syndrome*	Reported risk of T2D for a change from one category to the next, so the results could not be included in analysis.

REFERENCES:

.1 Wu F, Juonala M, Pahkala K, Buscot M-J, Sabin MA, Pitkänen N, et al. Youth and long-term dietary calcium intake with risk of impaired glucose metabolism and type 2 diabetes in adulthood. The Journal of Clinical Endocrinology & Metabolism. 2019;104(6):2.74-067

.2 Iwasaki Y, Arisawa K, Katsuura-Kamano S, Uemura H, Tsukamoto M, Kadomatsu Y, et al. Associations of nutrient patterns with the prevalence of metabolic syndrome: Results from the baseline data of the Japan multi-institutional collaborative cohort study. Nutrients. 2019;11(5):990.

.3 Benson VS, VanLeeuwen JA, Taylor J, Somers GS, McKinney PA, Van Til L. Type 1 diabetes mellitus and components in drinking water and diet: a population-based, case-control study in Prince Edward Island, Canada. J Am Coll Nutr. 2010;29(6):612-24.

.4 Liu S, Song Y, Ford ES, Manson JE, Buring JE, Ridker PM. Dietary calcium, vitamin D, and the prevalence of metabolic syndrome in middle-aged and older US women. Diabetes care. 2005;28(12):2926-32.

.5 Palacios C, Pérez CM ,González-Sepúlveda L, Corsino L, Albrecht SS, Siega-Riz AM, et al. Vitamin D, Calcium, Magnesium, and Potassium Consumption and Markers of Glucose Metabolism in the Hispanic Community Health Study/Study of Latinos. J Am Coll Nutr. 2020:1-10.

.6 Rogers IS ,Gunnell D, Emmett PM, Glynn LR, Dunger DB, Holly JM. Cross-sectional associations of diet and insulin-like growth factor levels in 7-to 8-year-old children. Cancer Epidemiology and Prevention Biomarkers. 2005;14(1):204-12.

.7 Kong SH, Kim JH, Hong AR, Cho NH, Shin CS. Dietary calcium intake and risk of cardiovascular disease, stroke, and fracture in a population with low calcium intake. The American journal of clinical nutrition. 2017;106(1):27-34.

.8 Samara A, Herbeth B, Ndiaye NC, Fumeron F, Billod S ,Siest G, et al. Dairy product consumption, calcium intakes, and metabolic syndrome–related factors over 5 years in the STANISLAS study. Nutrition. 2013;29(3):519-24.

.9 Takaya J, Yamanouchi S, Kino J, Tanabe Y, Kaneko K. A calcium-deficient diet in dams during gestation increases insulin resistance in male offspring. Nutrients. 2018;10(11):1745.

.10 Jeon J, Jang J, Park K. Effects of consuming calcium-rich foods on the incidence of type 2 diabetes mellitus. Nutrients. 2019;11(1):31.

.11 FENG L, Hu Z, Wang Q. Dietary calcium and serum 25OHD protect Chinese women from type 2 diabetes. J Nutr Sci Vitaminol. 2017;63(4):222-7.

.12 Woo HW, Lim Y-H, Kim MK, Shin J, Lee Y-H, Shin DH, et al. Prospective associations between total, animal, and vegetable calcium intake and metabolic syndrome in adults aged 40 years and older. Clin Nutr. 2020;39(7):2282-91.

.13 Moore-Schiltz L, Albert JM, Singer ME, Swain J, Nock NL. Dietary intake of calcium and magnesium and the metabolic syndrome in the National Health and Nutrition Examination (NHANES) 2001–2010 data. Br J Nutr. 2015;114(6):924-35.

.14 Kim K-N, Oh S-Y, Hong Y-C. Associations of serum calcium levels and dietary calcium intake with incident type 2 diabetes over 10 years: the Korean Genome and Epidemiology Study (KoGES). Diabetology & metabolic syndrome. 2018;10(1):1-7.

.15 Fumeron F, Lamri A, Abi Khalil C, Jaziri R, Porchay-Baldérelli I, Lantieri O, et al. Dairy consumption and the incidence of hyperglycemia and the metabolic syndrome: results from a French prospective study, Data from the Epidemiological Study on the Insulin Resistance Syndrome (DESIR). Diabetes care. 2011;34(4):813-7.

Supplemental Table 4. *Details of quality assessment of included studies in the systematic review and meta-analysis based on Newcastle-Ottawa Scale[1]. A. Cohort studies. B. Cross-sectional studies.*

A. ***Cohort studies.***

	Representativeness of the exposed cohort	*Selection of the non-exposed cohort*	*Ascertainment of exposure*	*Demonstration that outcome of interest was not present at start of study*	*Comparability of cohorts on the basis of the design or analysis*	*Assessment of outcome*	*Was follow-up long enough for outcomes to occur*	*Adequacy of follow up of cohorts*	*Total score*
Talaei et al, (2018)	*	*	*	*	**	*	*	*	*9*
Beydoun et al, (2018)	*	*		*	**	*	*		*7*
Oh et al, (2017)	*	*	*	*	**	*	*		*8*
Gagnon et al, (2011)	*	*	*	*	*	*	*		*7*
Kirii et al, (2009)	*	*	*	*	**	*	*		*8*
Villegas et al, (2009)	*	*	*	*	**	*	*	*	*9*
Van Dam et al, (2006)	*	*	*	*	**	*	*	*	*9*
Pittas et al, (2006)	*	*	*	*	**	*	*		*8*

B. ***Cross-sectional studies***

	Representativeness of the sample	*Sample size*	*Non-respondents*	*Ascertainment of the exposure (risk factor)*	*Comparability of subjects in different outcome groups*	*Assessment of outcome*	*Statistical test*	*Total score*
Shah et al, (2021)	*	*		**	**	*	*	*8*
Aritic et al, (2018)			*	*	**	**	*	*7*
Pannu et al, (2017)	*	*	*	*	**	**	*	*9*
Shin et al, (2016)	*	*	*	**	**	**	*	*10*
Shin et al, (2015)	*	*		**	**	**	*	*9*
Ferreira et al, (2013)		*	*	**	**	**	*	*9*
Kim et al, (2012)	*	*	*	**	**	**	*	*10*
Torres et al, (2011)			*	*	**	**	*	*7*
Torres et al, (2011)				**	**	**	*	*7*

[1]Wells GA, Shea B, O'Connell D, Peterson J, Welch V, Tugwell P. The Newcastle-Ottawa Scale (NOS) for Assessing the Quality of Nonrandomised Studies in Meta-Analyses. Available from: http://www.ohri.ca/programs/clinical_epidemiology/oxford.asp

Supplementary Table 5. GRADE evidence profile for dietary Ca intake in relation to T2D and hyperglycemia

Certainty assessment							*№ of patients*		*Effect*		*Certainty*	*Importance*
№ of studies	***Study design***	***Risk of bias***	***Inconsistency***	***Indirectness***	***Imprecision***	***Other considerations***	***Participants***	***Case***	***Relative (95% CI)***	***Absolute (95% CI)***		
7	Cohort studies	Not serious[a]	Not serious[b]	Not serious	Serious[c]	Dose response gradient	265145	14680 (5.5%)	**RR 0.82** (0.74 to 0.92)	**10 fewer per 1,000** (from 14 fewer to 4 fewer)	⨁⨁⨁⨁ High	CRITICAL
8	Cross-sectional studies	Not serious[d]	Not serious[e]	Not serious	Serious[f]	None	44001	4826 (11.0%)	**OR 0.88** (0.73 to 1.06)	**12 fewer per 1,000** (from 27 fewer to 6 more)	⨁⨁⨁◯ Moderate	CRITICAL

CI: confidence interval; **RR:** risk ratio; **OR:** odds ratio

a. Serious risk of bias; since, our quality assessment was based on Newcastle-Ottawa Scale. However, the effect size in the subgroup of studies with high quality was the same with the main analysis (RR: 0.82, 95%CI 0.73, 0.92; n=8). Not downgraded.

b. Serious inconsistency since I^2=53.6%. However, value of I2 was <50% in the subgroup of studies with higher than 50 years old population, significant, direction, and magnitude of the effect remained unchanged (RR: 0.76, 95%CI 0.70, 0.82; n=7, I^2=0.0%). Not downgraded.

c. Serious imprecision; since, 95%CI contained minimal value of 0.75.

d. Serious risk of bias; since, our quality assessment was based on Newcastle-Ottawa Scale. However, the effect size in the subgroup of studies with high quality was the same with the main analysis (OR: 0.92, 95%CI 0.77, 1.10; n=8). Not downgraded.

e. Serious inconsistency since I^2=69.5%. However, value of I^2 was <50% in the subgroup of studies with high quality, significant, direction, and magnitude of the effect remained unchanged (OR: 0.92, 95%CI 0.77, 1.10); n=8, I^2=46.4%). Not downgraded.

f. Serious imprecision; since, 95%CI contained minimal value of 0.75.

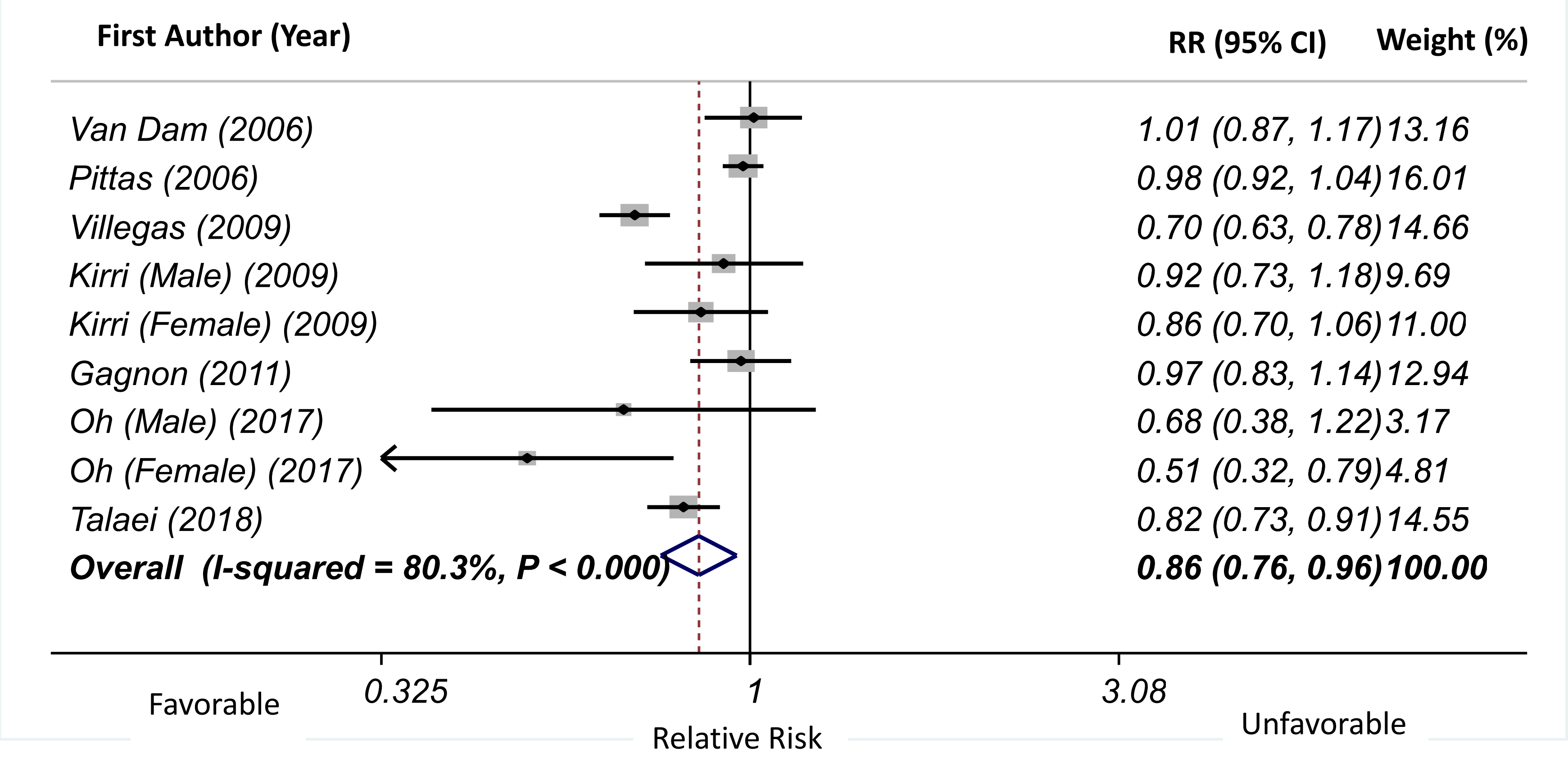

Supplemental Figure 1. Forest plots of linear dose-response meta-analysis of the association between each 600 mg/day increment in dietary Ca intake levels and T2DM in prospective cohort studies with representative adult populations.

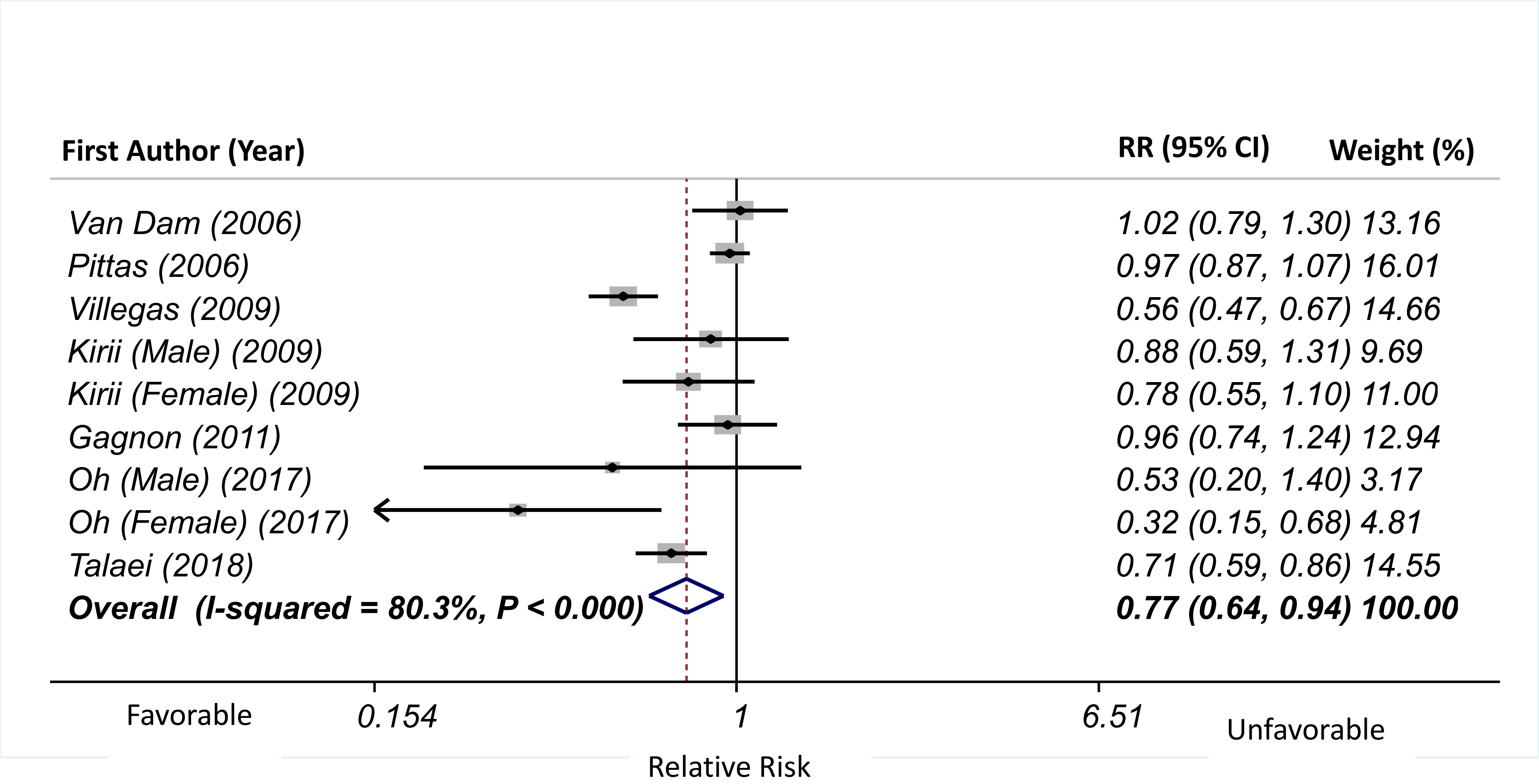

Supplemental Figure 2. Forest plots of linear dose-response meta-analysis of the association between each 1000 mg/day increment in dietary Ca intake levels and T2DM in prospective cohort studies with representative adult populations.

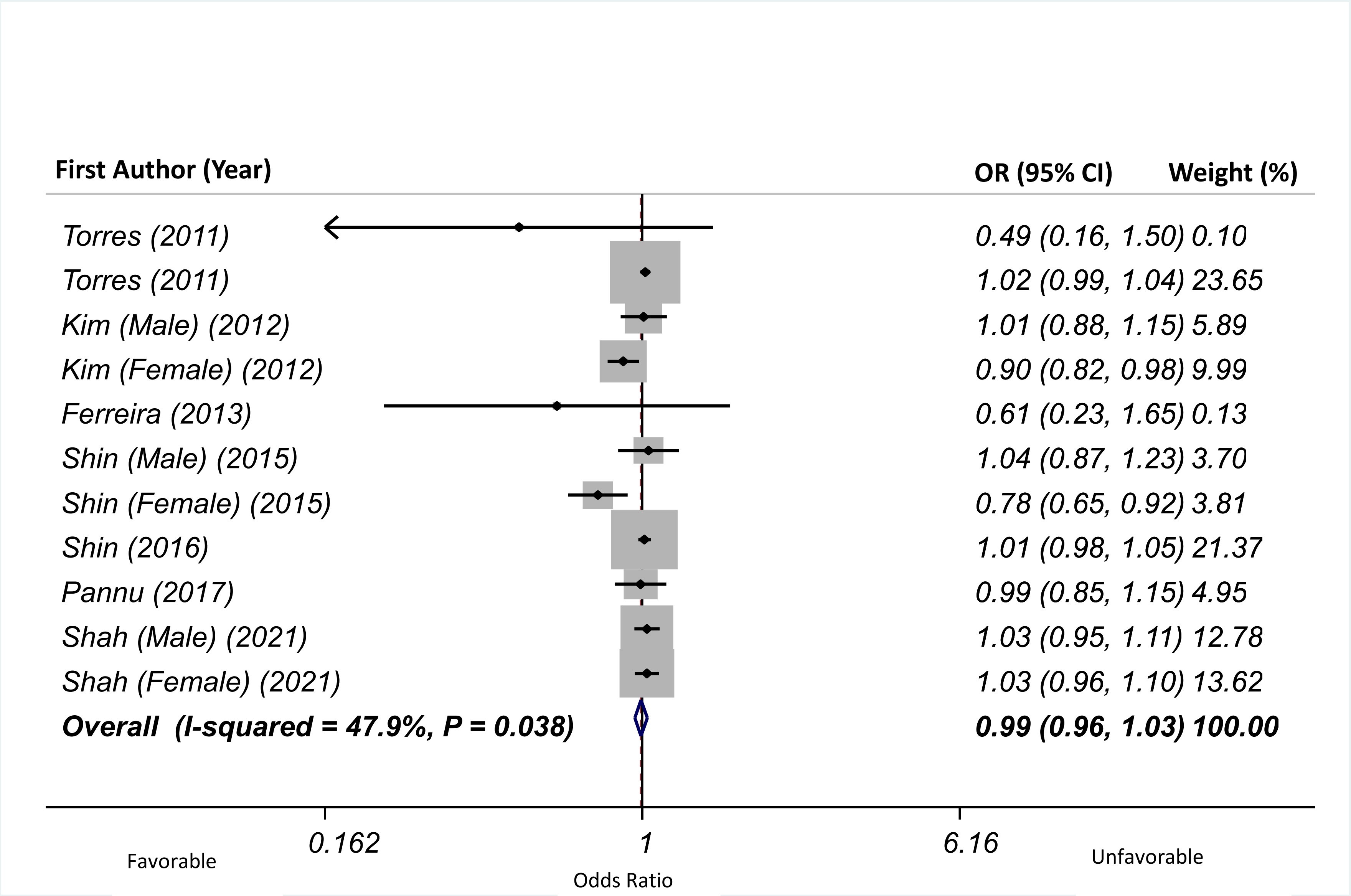

Supplemental Figure 3. Forest plots of linear dose-response meta-analysis of the association between each 300 mg/day increment in dietary Ca intake levels and T2DM/ hyperglycemia in cross-sectional studies.

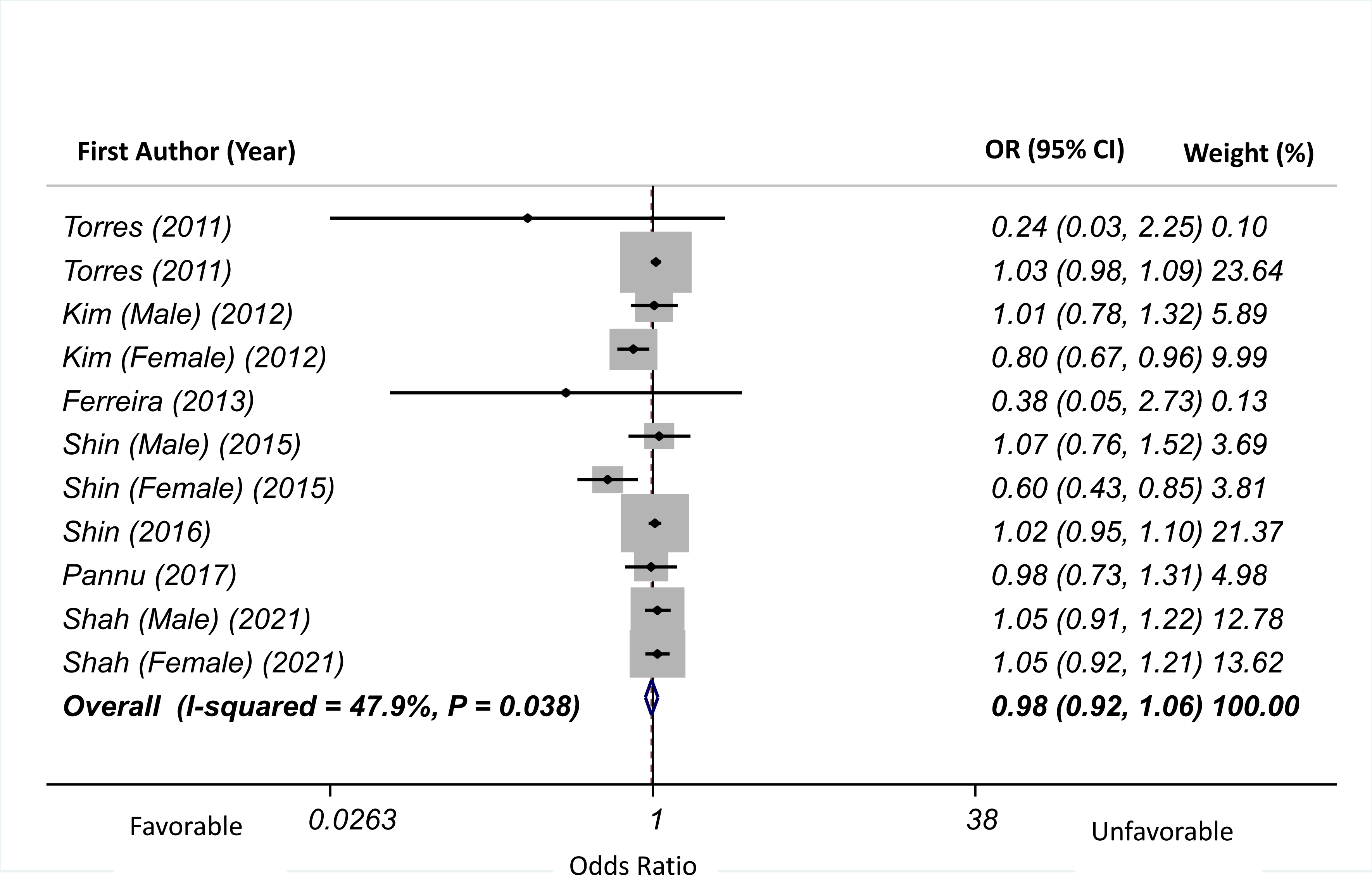

Supplemental Figure 4. Forest plots of linear dose-response meta-analysis of the association between each 600 mg/day increment in dietary Ca intake levels and T2DM/ hyperglycemia in cross-sectional studies.

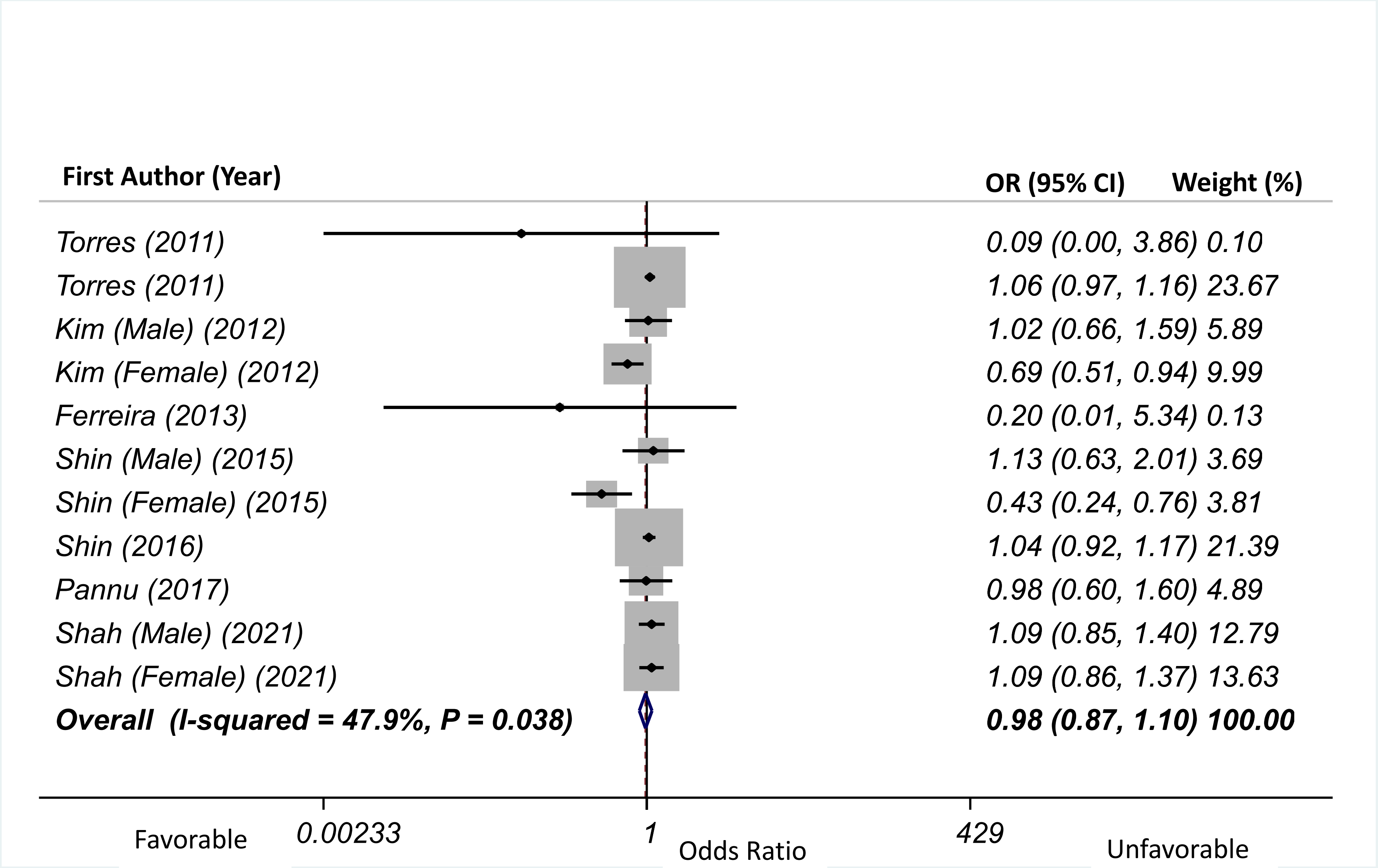

Supplemental Figure 5. Forest plots of linear dose-response meta-analysis of the association between each 1000 mg/day increment in dietary Ca intake levels and T2DM/ hyperglycemia in cross-sectional studies.

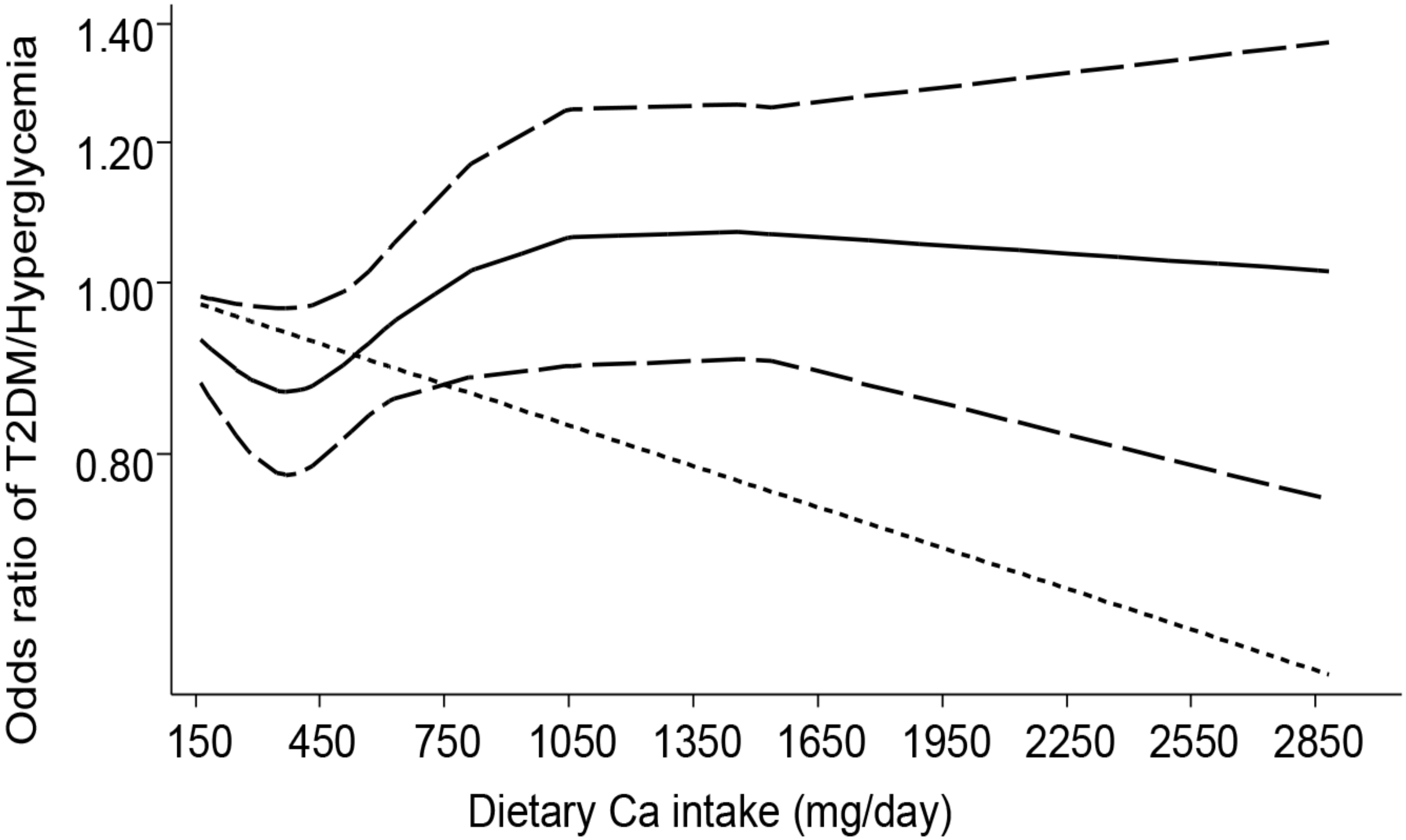

Supplemental Figure 6. Non-linear dose–response association between dietary Ca intake levels and T2DM/ hyperglycemia in cross-sectional studies. - - - , Linear model; ____, spline model.
